# Demographic patterns of two related desert shrubs with overlapping distributions in response to past climate changes

**DOI:** 10.3389/fpls.2024.1345624

**Published:** 2024-02-21

**Authors:** Qiushi Yu, Jing Hu, Xiaoke Hu, Yongfeng Zhou, Fanglin Wang, Shengxiu Jiang, Yuqi Wang

**Affiliations:** ^1^ Xinglongshan Forest Ecosystem National Positioning Observation and Research Station, Gansu Research Academy of Forestry Science and Technology, Lanzhou, China; ^2^ State Key Laboratory Breeding Base of Desertification and Aeolian Sand Disaster Combating, Gansu Desert Control Research Institute, Lanzhou, China; ^3^ Key Laboratory of Biodiversity Formation Mechanism and Comprehensive Utilization of the Qinghai-Tibet Plateau in Qinghai Province, Qinghai Normal University, Xining, China; ^4^ Guangdong Laboratory of Lingnan Modern Agriculture, Agricultural Genomics Institute at Shenzhen, Chinese Academy of Agricultural Sciences, Shenzhen, China

**Keywords:** population dynamics, regional expansion, multiple refugia, *Nitraria tangutorum*, *Nitraria sphaerocarpa*

## Abstract

Numerous studies have revealed that past geological events and climatic fluctuations had profoundly affected the genetic structure and demographic patterns of species. However, related species with overlapping ranges may have responded to such environmental changes in different ways. In this study, we compared the genetic structure and population dynamics of two typical desert shrubs with overlapping distributions in northern China, *Nitraria tangutorum* and *Nitraria sphaerocarpa*, based on chloroplast DNA (cpDNA) variations and species distribution models. We sequenced two cpDNA fragments (*trn*H-*trn*A and *atp*H-*atp*I) in 633 individuals sampled from 52 natural populations. Twenty-four chlorotypes, including eight rare chlorotypes, were identified, and a single dominant haplotype (H4) widely occurred in the entire geographical ranges of the two species. There were also a few distinctive chlorotypes fixed in different geographical regions. Population structure analyses suggested that the two species had significantly different levels of total genetic diversity and interpopulation differentiation, which was highly likely correlated with the special habitat preferences of the two species. A clear phylogeographic structure was identified to exist among populations of *N. sphaerocarpa*, but not exist for *N. tangutorum*. The neutral tests, together with the distribution of pairwise differences revealed that *N. tangutorum* experienced a sudden demographic expansion, and its expansion approximately occurred between 21 and 7 Kya before present, while a rapid range expansion was not identified for *N. sphaerocarpa*. The ecological niche modeling (ENM) analysis indicated that the potential ranges of two species apparently fluctuated during the past and present periods, with obvious contraction in the Last Glacial Maximum (LGM) and recolonization in the present, respectively, comparing to the Last Interglacial (LIG). These findings suggest that the two species extensively occurred in the Northwest of China before the Quaternary, and the current populations of them originated from a few separated glacial refugia following their habitat fragmentation in the Quarternary. Our results provide new insights on the impact of past geological and climatic fluctuations on the population dynamics of desert plants in northwestern China, and further enforce the hypothesis that there were several independent glacial refugia for these species during the Quaternary glaciations.

## 1 Introduction

It is believed that past geological events and climate oscillations have played important roles in promoting geographical distribution and population structure of species (Bennett, 1997; Abbott et al., 2000; Hewitt, 2000; 2004; Avise, 2004; Wu et al., 2010). The uplift of the Qinghai-Tibet Plateau (QTP) accelerated the aridification and desert expansion in the Northwest of China (Sun et al., 1998; Guo et al., 2002; Sun and Liu, 2006). Some studies showed that deserts in North China began to form in the Pliocene (Sun et al., 1998; Yang et al., 2006), and further expanded considerably in the Quaternary glacial periods (Bush et al., 2004). The formation and subsequent expansion/contraction fluctuations of these deserts corresponding to climate fluctuations seemed to have resulted in the range fragmentation, subdivision and diversification of some desert plants in North China (Li et al., 2012; Yu et al., 2013; Meng et al., 2015; Qian et al., 2016; Zhang et al., 2017; Hu et al., 2022). Furthermore, the glacial and interglacial cycles in the Quaternary also probably accelerated range fragmentation, vicariance, and regional-scale differentiation of the desert plants in this area (Meng et al., 2015; Zhang et al., 2017). Actually, how these desert plants in North China responded to past environmental changes remains still unclear.

Numerous studies revealed that past geological events usually caused concordant demographic patterns in sympatric species (e.g. Hewitt, 2000; 2004; Haring et al., 2007; Kirchman and Franklin, 2007). However, a few studies found that some species with overlapping distribution might respond differently to similar historical events, resulting in species-specific population structure and demographic patterns (e.g. Taberlet et al., 1998; Zink et al., 2001; Polihronakis and Caterino, 2010; Zhang et al., 2012). The different responses of sympatric plants to the similar historical events may be induced by different factors. First, the more recent range expansion usually results in shallow differentiation among populations, because the accumulation of genetic variations and lineage sorting in different ranges require a long historical process in nature (Zink, 1996; Conroy and Cook, 2000; Hewitt, 2000; 2004; Polihronakis and Caterino, 2010). Second, habitat preferences might affect gene flow between populations/species of plants with similar dispersal ability, and further contribute to genetic divergence (Michaux et al., 2005; Hamer and McDonnell, 2010; Lange et al., 2010; Morris-Pocock et al., 2010; Yu et al., 2013). Third, plants with high ecological plasticity and broad ranges have more opportunities to survive and disperse when in unfavorable conditions than other plants with limited niches and narrow ranges (Zhang et al., 2012). For the desert plants with high drought-tolerance and different habitat preferences in North China, it is desirable to know how their phylogeographical structures and population dynamics responded to climatic oscillations, and whether they had multiple ice age refugia in the Quaternary.

*Nitraria* L. (Zygophyllaceae), known as a ‘living fossil’ of historical flora, is a genus of Tertiary relic shrubs widely distributed in Central Asia, Southeast of Europe, Mongolia and China, North Africa, and Australia (Liu, 1999; Pan et al., 2003; Temirbayeva and Zhang, 2015). It consists of 13 species or so, including some primitive diploid species and a few evolved tetraploid taxa (Pan et al., 2003). Out of these related desert species, eight occurs in arid or semi-arid area of Northwest China, including Xinjiang, northern Qinhai, Hexi corridor in Gansu, Ningxia, northern Shanxi, central and western Inner Mongolia, and a narrow distribution in western Sichuan (Pan et al., 2003; Yang, 2006; Temirbayeva and Zhang, 2015). These species play important roles in maintaining the local ecosymtem balance and fixing flowing sand, and their fruits are edible for eating (Pan et al., 1999). A few studies revealed that the genus originated from eastern Central Asia, including northern China and Mongolia, and subsequently dispersed to Africa, western Central Asia, and Australia (Temirbayeva and Zhang, 2015; Zhang et al., 2015). Actually, the eastern Central Asia contains some endemic species and ancient diploid species (*N. sphaerocarpa*) as well as young evolved tetraploid species (Cain, 1994), indicating that the region is an indisputable hotspot and/or diversification center of this genus (Pan et al., 1999, 2003; Yang, 2006; Temirbayeva and Zhang, 2015). As ancient plants in this hotspot region, there are some attractive and unclear issues that how these species responded to the past geological and climatic changes, and the current populations of these species originated from several independent glacial refugia or the same refugium. Especially, we are interested to know that if these sympatric related species differently responded to the past climatic oscillations, particularly aridification and desert expansion/contraction.

Among the eight *Nitraria* species distributed in Northwestern China, three species, i.e. *N*. *pamirica*, *N. sinesis*, and *N. schoberi*, only occur in some independent or narrow geographical regions, for example, *N*. *pamirica* occurs independently in Pamirs in Xinjiang, *N. schoberi* is only limited to the Junggar Basin in northern Xinjiang, and *N. sinesis* only grows on the sands along the sea of the Liaodong Peninsula which is far from the core distribution area of other *Nitraria* species in China (Xu et al., 1998; Pan et al., 1999; Pan et al., 2003; Temirbayeva and Zhang, 2015). The other five species, i.e. *N. praevisa*, *N. sphaerocarpa*, *N. sibirica*, *N. tangutorum*, and *N. roborowskii*, share the overlapping distributions in Northern China. However, we have not observed any different habitat preferences among these species. Actually, *N. praevisa* is a disputable species in classification (Xu et al., 1998; Pan et al., 1999; Pan et al., 2003). In addition, these species differentiated lately and their interspecific delimitation based on morphological differentiation is relatively blurred. Therefore, the two species, *N. tangutorum* and *N. sphaerocarpa*, provide us with ideal materials for comparatively studying demographic history of sympatric desert species in response to past climate changes.

*N. tangutorum* and *N. sphaerocarpa* widely grow on gravel Gobi or sandy land in northern China (Pan et al., 2003), and have strong resistance to drought, soil salinization and flowing sand. The two species constantly play vital roles in maintaining local ecosystem stability in desert regions (Li et al., 2005; Shi et al., 2014). The fruits of *N. tangutorum* are rich in various vitamins and amino acids as well as essential mineral elements, and therefore have high nutritional and health benefits (Liu et al., 2016; Wang et al., 2018). The two species can reproduce through clonal ramets under natural conditions and form nebkhas like small islands in desert. *N. sphaerocarpa* is the most primitive diploid species of this genus, and probably differentiated from other *Nitraria* species in Paleocene (Zhang et al., 2015). It is characterized by vesicular drupes with dry and membranous exocarp, blade linear to oblanceolate-linear leaves (Xu et al., 1998), which is significantly different from other *Nitraria* species. However, *N. tangutorum*, endemic to northern China (eastern Central Asia), is a relatively evolved tetraploid taxon, and diverged from other *Nitraria* species in late Miocene approximately (Zhang et al., 2015). It has wide distributional ranges in northern China, including Xinjiang, northern Qinghai, Hexi corridor in Gansu, Ningxia, and the central and western regions of Inner Mongolia. This species is significantly different from *N. sphaerocarpa* due to having fleshy drupes and broader leaves (Xu et al., 1998). The two species mainly disperse through seeds which are probably carried by different animals (e.g. mice, birds). However, the berry-like fruits of *N. tangutorum* are more favored by animals for consumption than *N. sphaerocarpa*, which may have caused longer distance dispersal of *N. tangutorum* seeds than *N. sphaerocarpa* (Zhang et al., 2015). In addition, the breeding system of the two species is dominantly xenogamous, but depends on pollinators (Li et al., 2013), indicating that the pollen dispersal ability of them is extremely limited. Although the two species shared a few locations in their geographical ranges, we found that they have contrasting habitat preferences: *N. sphaerocarpa* prefer to grow on gravel sandy land or Gobi land in western Inner Mongolia, Hexi corridor of Gansu province, and Xinjiang, while *N. tangutorum* mainly grows on shifting sandy land or semi-fixed sandy land in North China.

Chloroplast DNA (cpDNA) is generally maternally inherited in angiosperms (Palmé et al., 2003) and has been widely used to revealing glacial refugia and postglacial recolonization patterns of plant species (e.g., Liu et al., 2009; Wu et al., 2010; Zhang et al., 2017). The ecological niche modeling (ENM) on the basis of maximum entropy modeling (Phillips et al., 2004, 2006) is also a good method for predicting species geographic distributions and historical dynamics with presence-only data (Yin et al., 2015; Qian et al., 2016; Zhang et al., 2017). In this study, we used two cpDNA fragments (*trn*H-*psb*A and *atp*H-*atp*I) and the ENM method to compare genetic structure and demographic patterns of the two sympatric desert shrubs. We aimed to address the following questions: (1) how did past geological and climatic fluctuations affect the genetic structure and lineage differentiation of the two species? (2) Did the two species experience apparent range expansion/contraction during the different historical periods (e.g. LIG, LGM), and were there several independent glacial refugia (multiple refugia hypothesis) for *Nitraria* species in northern China during the Quaternary glaciations? And (3) are there any differences of population structure and lineage differentiation between the two species? This study would further shed light on the geographical subdivision, demographic patterns of desert species in northern China in response to past geological and climatic changes, i.e. aridification, desert formation and expansion.

## 2 Materials and methods

### 2.1 Population sampling

**Figure 1:**
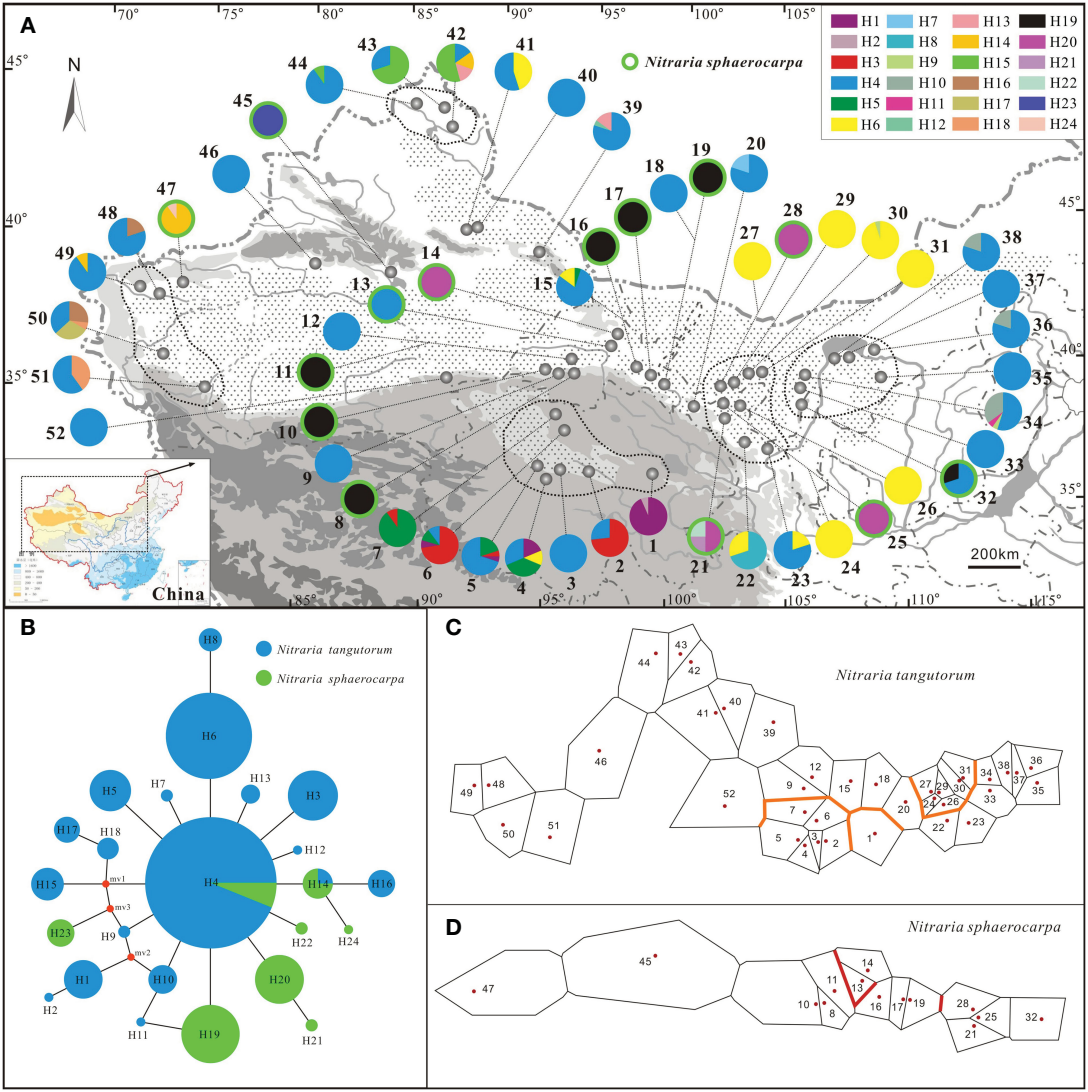
Sampling locations, geographic distribution of the chlorotypes, the phylogenetic network, and genetic barriers among populations of *Nitraria tangutorum* and *Nitraria sphaerocarpa*. **(A)** Sampling locations and chlorotype frequencies in surveyed populations. Populations with green circle belong to *N. sphaerocarpa*, while other populations without green circle belong to *N. tangutorum*. **(B)** Phylogenetic network of the twenty-four chlorotypes detected in the study. Circle size is proportional to the frequency of a chlorotype over all populations, with the largest circle representing the most abundant chlorotype. The small red dots represent median vectors (i.e. unsampled or extinct chlorotypes). **(C, D)** Genetic barriers to chlorotypes between different surveyed populations for *N. tangutorum* and *N. sphaerocarpa*, respectively.

A total of 633 individuals were sampled from 52 natural populations of *N. sphaerocarpa* and *N. tangutorum* in the study, including 119 individuals from 14 populations of *N. sphaerocarpa*, and 514 individuals from 38 populations of *N. tangutorum*. These populations almost cover the whole geographical ranges of the two species except a few populations of *N. sphaerocarpa* occurring in Mongolia (**Table 1**; **Figure 1A**). However, this will not bring a significant influence on the inference of the population structure of the two species, since these populations in Mongolia just occur on the fringe of the whole distribution of *N. sphaerocarpa* and represent only a small part of the gene pool in this study. In the sampled populations, five population sites are overlapped for the two species, for example populations 11 of *N. sphaerocarpa* and 12 of *N. tangutorum* share the same site, and 15 and 16 share another site (**Table 1**; **Figure 1A**). In each population, fresh leaves were collected from 5-30 individuals at least 100 meters apart and rapidly dried with silica gel in the field. The site information of each population, including latitude, longitude and altitude, were recorded using an Etrex GIS monitor (**Table 1**). In addition, we collected a voucher specimen for each population, and some seeds and flowers for further studies on morphology and germination.

**Table 1:** Sampling sites, sample size, genetic diversity estimates, and haplotype distribution for 52 populations of *Nitraria tangutorum* and *Nitraria sphaerocarpa*.

Pop code	Species	Sample site	Latitude (°N)	Longitude (°E)	Elevation (m)	Specimen code^*^	Sample size	Haplotypes (Number)	*H*_E_
1	T	Wulan, QH	36° 46’ 17’’	98° 56’ 26’’	3069	19072901	15	H1(14); H2(1)	0.133
2	T	Luomuhong, QH	36° 22’ 15’’	96° 05’ 34’’	2768	19073002	15	H3(11); H4(4)	0.419
3	T	Germu, QH	36° 22’ 23’’	95° 36’ 17’’	2816	19073101	10	H4(10)	0.000
4	T	Jinaigou, QH	36° 08’ 02’’	94° 48’ 26’’	3162	22072301	16	H1(3); H4(5); H5(6); H6(2)	0.758
5	T	GermuX, QH	36° 24’ 45’’	94° 27’ 33’’	2796	19073001	20	H1(1); H3(1); H4(14); H5(4)	0.490
6	T	Xiaochaidan, QH	37° 35’ 42’’	95° 32’ 44’’	3198	19080201	29	H1(2); H3(21); H4(3); H5(3)	0.466
7	T	Dachaidan, QH	38° 08’ 16’’	94° 50’ 07’’	3161	19080202	10	H3(1); H5(9)	0.200
8	S	Dunhuang, GS	39° 40’ 14’’	94° 43’ 20’’	1870	19080303	10	H19(10)	0.000
9	T	Subei,GS	39° 34’ 23’’	94° 45’ 36’’	2079	19080302	10	H4(10)	0.000
10	S	Akesai, GS	39° 37’ 03’’	94° 19’ 35’’	1700	19080301	5	H19(5)	0.000
11	S	DunhuangB, GS	40° 15’ 25’’	95° 16’ 41’’	1093	19080501	6	H19(6)	0.000
12	T	DunhuangB, GS	40° 15’ 25’’	95° 16’ 41’’	1093	19080502	10	H4(10)	0.000
13	S	Yumen, GS	40° 46’ 45’’	96° 40’ 11’’	1549	19080503	10	H4(10)	0.000
14	S	Yinaoxia, GS	41° 16’ 34’’	96° 56’ 53’’	1948	19080504	10	H20(10)	0.000
15	T	Majiadi, GS	40° 00’ 03’’	97° 30’ 27’’	1699	19080601	20	H4(16); H5(1); H6(3)	0.353
16	S	Majiadi, GS	40° 00’ 03’’	97° 30’ 27’’	1699	19080602	8	H19(8)	0.000
17	S	Jinta, GS	39° 52’ 29’’	98° 43’ 22’’	1363	19080603	10	H19(10)	0.000
18	T	Gaotai, GS	39° 48’ 56’’	99° 01’ 53’’	1336	19080701	10	H4(10)	0.000
19	S	Gaotai, GS	39° 48’ 56’’	99° 01’ 53’’	1336	19080702	4	H19(4)	0.000
20	T	Zhangye, GS	38° 44’ 18’’	100° 46’ 42’’	1705	19072701	10	H4(8); H7(2)	0.356
21	S	Jinchang, GS	38° 34’ 03’’	102° 16’ 04’’	1456	19080801	8	H20(4); H21(2); H22(2)	0.714
22	T	Gulang, GS	37° 39’ 14’’	103° 11’ 05’’	1741	19091601	10	H6(3); H8(7)	0.467
23	T	Jingtai, GS	37° 27’ 42’’	104° 26’ 11’’	1730	19093001	10	H4(8); H6(2)	0.356
24	T	MinqinHSG, GS	38° 59’ 31’’	102° 28’ 32’’	1438	19091801	12	H6(12)	0.000
25	S	MinqinHSG, GS	38° 59’ 31’’	102° 28’ 32’’	1438	19091802	8	H20(8)	0.000
26	T	MinqinXSW, GS	38° 35’ 12’’	102° 58’ 31’’	1372	19091701	19	H6(19)	0.000
27	T	YouqiSM, IM	39° 22’ 35’’	102° 13’ 33’’	1583	21062501	10	H6(10)	0.000
28	S	YouqiSM, IM	39° 22’ 35’’	102° 13’ 33’’	1583	21062502	10	H20(10)	0.000
29	T	YouqiYBL, IM	39° 18’ 11’’	102° 43’ 36’’	1240	19091803	15	H6(15)	0.000
30	T	ZuoqiBY, IM	40° 04’ 50’’	103° 56’ 05’’	1378	19091805	20	H6(19); H9(1)	0.100
31	T	YouqiAB, IM	40° 10’ 28’’	104° 02’ 39’’	1415	21062503	10	H6(10)	0.000
32	S	ZuoqiXJZ, IM	38° 54’ 44’	105° 41’ 01’’	1531	19091901	10	H4(7); H19(3)	0.467
33	T	ZuoqiJLT, IM	39° 24’ 59’’	105° 40’ 51’’	1133	19091902	10	H4(10)	0.000
34	T	ZuoqiND, IM	40° 07’ 19’’	105° 42’ 13’’	1078	19091903	20	H4(11); H9(1); H10(7); H11(1)	0.600
35	T	Balagong, IM	39° 53’ 42’’	108° 28’ 01’’	1246	19092101	10	H4(10)	0.000
36	T	Jixiang, IM	40° 48’ 59’’	108° 07’ 38’’	1038	19092002	10	H4(8); H10(2)	0.356
37	T	Huhemudu, IM	40° 30’ 35’’	107° 16’ 14’’	1039	19092001	10	H4(10)	0.000
38	T	Dengkou, IM	40° 29’ 17’’	106° 43’ 24’’	1034	19091904	10	H4(8); H10(2)	0.356
39	T	Balikun, XJ	43° 37’ 08’’	93° 01’ 42’’	1604	21072701	20	H4(16); H12(1); H13(3)	0.353
40	T	Qitai, XJ	44° 25’ 16’’	90° 06’ 05’’	673	21080101	10	H4(10)	0.000
41	T	Changji, XJ	44° 11’ 18’’	89° 34’ 13’’	653	21073102	20	H4(11); H6(9)	0.521
42	T	Jiaosate, XJ	47° 15’ 18’’	88° 11’ 11’’	564	21080203	13	H4(2); H13(2); H14(2); H15(7)	0.692
43	T	WoyimaK, XJ	47° 44’ 34’’	87° 32’ 11’’	513	21080301	10	H4(3); H15(7)	0.467
44	T	Jimunai, XJ	47° 45’ 48’’	86° 06’ 55’’	541	21080401	10	H4(9); H15(1)	0.200
45	S	Yanqi, XJ	42° 00’ 10’’	86° 16’ 19’’	1072	22071701	10	H23(10)	0.000
46	T	Baicheng, XJ	41° 51’ 00’’	82° 46’ 58’’	1312	22071601	10	H4(10)	0.000
47	S	AtushiHLJ, XJ	40° 15’ 03’’	77° 09’ 44’’	1618	22071408	10	H14(9); H24(1)	0.200
48	T	AtushiSTK, XJ	39° 46’ 14’’	76° 17’ 39’’	1254	22071407	10	H4(8); H16(2)	0.356
49	T	Wuqia, XJ	39° 44’ 29’’	75° 29’ 14’’	2299	22071402	10	H4(9); H14(1)	0.200
50	T	Yecheng, XJ	37° 18’ 43’’	77° 08’ 40’’	2156	22071101	30	H4(11); H16(8); H17(9); H18(2)	0.724
51	T	Hetian, XJ	36° 38’ 44’’	79° 52’ 05’’	1824	22071001	10	H4(6); H18(4)	0.533
52	T	Ruoqiang, XJ	38° 29’ 14’’	90° 06’ 51’’	3123	22070601	10	H4(10)	0.000
Total							633		0.208

T, *N. tangutorum*; S, *N. sphaerocarpa*; GS, Gansu; IM, Inner Mongolia, XJ, Xinjiang; QH, Qinghai; *H*_E_, haplotype diversity. ^*^ Voucher specimens have been deposited in herbarium of Gansu Desert Control Research Institute (Lanzhou, China).

### 2.2 DNA extraction, amplification and sequencing

Total genomic DNA was extracted using DNeasy Plant Mini Kits (Qiangen, Valencia, CA, USA). Ten samples from 10 geographically distant populations were selected for primer scanning with primer pairs of six cpDNA fragments, i.e. *trn*L*-trn*F, *rpl32*-*trn*L (Taberlet et al., 1991), *rp*S-*trn*K (Shaw et al., 2007), *trn*H-*psb*A, *atp*H-*atp*I, and *ndh*C-*trn*V (Dong et al., 2012; Qian et al., 2016). Two cpDNA fragments, *trn*H-*psb*A and *atp*H-*atp*I, were found to contain useful and appropriate polymorphic loci, and further used for surveying genetic variations of the two species in the present study. Polymerase chain reaction (PCR) amplification was performed in a Bio-Rad PCR system S1000 Thermal Cycler (USA). The reaction volume was 25 µL, containing 1.0 µL genomic DNA extract (about 20-40 ng DNA), 2.5 µL 10×PCR buffer, 0.2µL Taq DNA polymerase (5U/µL, TakaRa Biotech Co., Dalian, China), 0.5 mmol/L dNTPs, 1.5 mmol/L MgCl_2_, and 2µmol/L each primer. The thermal profile for *trn*H-*psb*A included the following phases: 94°C for 5 min, followed by 30 cycles at 94°C for 45 s, 56°C for 45 s, and 72°C for 105 s, then a final extension phase of 7 min at 72°C. The thermal profile for *atp*H-*atp*I was as follows: 94°C for 3 min, followed by 30 cycles at 94°C for 30 s, 58°C for 30 s, and 72°C for 60 s, then ended with an extension step of 5 min at 72°C. The PCR products were directly used for sequencing on an ABI 3130xl Genetic Analyzer platform (Applied Biosystems, USA), with forward and/or reverse primers. The obtained DNA sequences were aligned with Clustal_X (Thompson et al., 1997), and carefully revised manually. All the newly obtained sequences of *N. sphaerocarpa* and *N. tangutorum* were deposited in EMBL GenBank (https://www.ncbi.nlm.nih.gov/) under the accession numbers of OR801339-OR801365.

### 2.3 Haplotype diversity and population structure analysis

Nucleotide diversity parameters, including number of haplotypes, nucleotide diversity (Pi), number of polymorphic sites, were calculated using DnaSP 5.10 software (Librado and Rozas, 2009). We used NETWORK version 4.2.0.1 (Bandelt et al., 1999; available at http://www.fluxus-engineering.com) to construct Median-joining network of cpDNA haplotypes based on the data generated through DnaSP. We also reconstructed the phylogenetic tree among these haplotypes, using one individual of *Zygophyllum xanthoxylum* as outgroup. The neighbor-joining (NJ) tree, maximum-parsimony (MP) tree and maximum-likelihood (ML) tree, with bootstrap values of 1000 replicates (Felsenstein, 1985), were obtained respectively through MEGA 4 software (Tamura et al., 2007).

Unbiased genetic diversity (*H*_E_) was estimated for each population based on cpDNA haplotype composition (Nei, 1987). According to the methods described by Pons and Petit (1996), average gene diversity within populations (*H*s), total gene diversity (*H*_T_) and population differentiation between populations (*G*_ST_ and *N*_ST_) were calculated for the two species using the PERMUT program (available at http://www.pierroton.inra.fr/genetics/labo/Software/Permut/). *G*_ST_ depends only on the frequencies of the haplotypes while *N*_ST_ takes into account the similarity between haplotypes as well as haplotype frequencies (Pons and Petit, 1996). *G*_ST_ and *N*_ST_ were compared using the u-statistic to determine the presence of phylogeographic structure. A higher *N*_ST_ than *G*_ST_ denotes a significant phylogeographic structure, indicating that some closely related haplotypes usually occur in the same or nearby geographical regions (Pons and Petit, 1996). Analyses of AMOVA were carried out with Arlequin version 3.0 to estimate genetic differentiation within populations, between populations within groups and between groups, with significant tests of 1000 permutations (Excoffier et al., 2005). The correlation between geographic distance and genetic differentiation among populations was tested using the Mantel test through TFPGA version 1.3 (Mantel, 1967).

To further reveal spatial genetic structure of the two species, we performed a spatial analysis of molecular variance using SAMOVA version 1.0 (Dupanloup et al., 2002, http://web.unife.It/progetti/genetica/Isabelle/samova.html) based on the cpDNA haplotypes and geographical localities of all populations sampled in the study. In the analysis, the number of population groups (*K*) was defined using a simulated annealing approach, and *K* values were set between 2 and 12 with each simulation starting from 100 random initial conditions. A genetic differentiation index (*F*_CT_) among groups was calculated, and the optimal configuration of groups was determined using an iterative simulated annealing process (Wright, 1978). In addition, biogeographical boundaries or potential gene flow barriers among populations were examined with Monmonier’s maximum-difference algorithm in BARRIER v2.2 (Manni et al., 2004). The robustness of these barriers was evaluated through running BARRIER program on the basis of 100 replicates of population average pairwise difference matrices. The difference matrices in the test were obtained through bootstrapping of haplotype sequences in SEQBOOT (Felsenstein, 2005).

### 2.4 Population demographic analyses

Firstly, we carried out mismatch distribution analyses (Schneider and Excoffier, 1999) in DnaSP 5.10 version (Librado and Rozas, 2009) to test sudden demographic expansions of the two species. In general, populations with a sudden demographic expansion in the past should display a smooth and unimodal distribution (Slatkin and Hudson, 1991; Rogers and Harpending, 1992). Secondly, we performed Tajima’s *D* (Tajima, 1989) and Fu’s *F*s (Fu, 1997) statistical tests using Arlequin version 3.0 (Excoffier et al., 2005) to infer historical demographic expansions of *N. sphaerocarpa* and *N. tangutorum* respectively. The validity of the expansion model was determined with the sum of squared deviation (*SSD*) between the observed and expected mismatches. The significance of expansion model (Harpending, 1994) was tested using Harpending’s raggedness index and associated *P* values. We used the formula τ = 2*ut* (Rogers and Harpending, 1992) to estimate the expansion time (*t*). In the formula, τ is the mode of mismatch distribution defined with units of evolutionary time, and *u* is the mutation rate per generation, which was estimated through the relationship *u* = 2 *μkg*. Here *μ* refers to the substitution rate per nucleotide site per year (s/s/y), *k* is the sequence length, and *g* represents the generation time in years. The average sequence mutation rates of chloroplast DNA in angiosperms (1.0-3.0×10^-9^s/s/y) were used to estimate the expansion time of the species (Wolfe et al., 1987), since there was no fossil to calibrate cpDNA mutation rates of *Nitraria* species. According to our field observations and some cultivation experiments, the generation time of the two species was about 3 years. Thirdly, we performed ecological niche modeling (ENM) on the basis of maximum entropy approach to infer the potential distribution ranges of the two species in the LIG (ca. ~140Kya), the LGM (ca. ~21 Kya), the present, and the future (2050), using the MAXENT version 3.3.3k (Phillips et al., 2004; 2006; Peterson and Soberón, 2012). In the simulating analysis, a “maximum training sensitivity plus specificity” threshold was used to determine suitable/unsuitable habitat (Liu et al., 2010). A jackknife test was carried out to estimate the contributions of different bioclimatic variables to the prediction of the distributional models. The goodness of models was evaluated with the area under the receiver operating characteristic (ROC) curve (AUC scores) (Fawcett, 2006). In order to reduce effects of spatial autocorrelation, we deleted duplicates of records. A total of 128 distribution sites, including 84 sites for *N. tangutorum* and 44 sites for *N. sphaerocarpa*, were used in the ENM analyses. These sites consist of 52 sampling sites in the study and 76 specimen records from the Chinese Virtual Herbarium (CVH, http://www.cvh.org.cn/). All bioclimatic layers with 19 bioclimatic variables at a resolution of 30 arc seconds were obtained from the WorldClim database (available at http://www.worldclim.org/; Hijmans et al., 2005). In order to avoid multivariate collinearity of environmental variables, which could lead to model over-fitting, we only retained distinct sets of variables that contributed most to models, and eliminated one variable per pair with correlations of r ≥ 0.8 according to Pearson correlation value (Wan et al., 2016). Seven bioclimatic variables, i.e. precipitation of warmest quarter (Bio18), temperature seasonality (Bio4), precipitation of driest quarter (Bio17), annual mean temperature (Bio1), mean temperature of coldest quarter (Bio11), annual precipitation (Bio12), and precipitation seasonality (Bio15), were selected for modeling the potential range of the two *Nitraria* species. We used 30 replicates with 80% of the geographical sites for training and 20% for testing. Graphics for distribution models in different periods of the two species were drawn using DIVA-GIS 7.5.

## 3 Results

### 3.1 Chloroplast DNA variation and geographic distribution

A total of 23 single nucleotide substitutions and 2 indels of 3 or 6 nucleotides within *trn*H-*trn*A sequence were detected in 633 individuals sampled from 52 populations and the length of the cpDNA fragment was 834 bp. However, only 8 nucleotide substitutions were detected in the *atp*H-*atp*I cpDNA fragment. The total length of combined the two chloroplast DNA sequences was 1794 bp, comprising 24 parsimony informative sites. Haplotype diversity (*H*_d_) and nucleotide diversity (Pi) calculated with DnaSP 5.10 software were 0.7819 and 0.00094, respectively. A total of twenty-four different chlorotypes (H1-H24) were identified among all individuals sampled in the study (**Supplementary Table S1**). However, the relationships among these chlorotypes have not been resolved by phylogenic analysis (**Supplementary Figure S1**). Half of the populations sampled in this study were fixed for a single chlorotype, and the other half of the populations were polymorphic (**Table 1**, **Figure 1A**). The most common chlorotypes were H4, H6 and 19 (**Figures 1A, B**). H4 widely occurred in most populations of *N. tangutorum* at high frequency and a few populations of *N. sphareocarpa* (13 and 32). H6 only occurred in some populations of *N. tangutorum* as a single chlorotype (24, 26, 27, 29 and 31) or at different frequencies (4, 15, 22, 23, 30 and 41). However, H19 was only fixed for most populations of *N. sphaerocarpa* as a single chlorotype (8, 10, 11, 16, 17, and 19), and for one population (32) at lower frequency. The other haplotypes occurred in a few populations at different frequencies. Especially, there were eight rare haplotypes (H2, H7, H9, H11, H12, H21, H22 and H24) which occurred only in one or two individuals of the two species, and the other two rare haplotypes, H13 and H18, were present only in two populations of *N. tangutorum* at low frequencies, respectively (H13: 39, 42; H18: 50, 51). In addition, H4 and H14 were shared by two species while all the other haplotypes identified in this study were fixed by only one of the two species (**Figure 1B**). The haplotype diversity (*H*_E_) calculated for each population ranged from 0 to 0.758, with a mean value of 0.208 (**Table 1**). Population 4, which had the greatest haplotype diversity (*H*_E_ = 0.758), was polymorphic for chlorotypes H1, H4, H5, and H6. Furthermore, the other four populations (21, 34, 42 and 50) had high haplotype diversities (*H*_E_ = 0.714, 0.600, 0.692 and 0.724, respectively) for three or four haplotypes. Although the two populations (5 and 6) contained high haplotype numbers, their values of *H*_E_ were low (*H*_E_ = 0.490, and 0.466, respectively) because of low frequencies of some chlorotypes occurring in these populations (e.g., H1, H3). Notably, population pairs of *N. tangutorum* and *N. sphareocarpa* at the same sampling site contained completely different chlorotype composition (**Figure 1A**; population pairs 11/12, 15/16, 18/19, 24/25, and 27/28), implying that a complete reproductive isolation has been established between the two related species.

### 3.2 Population structure and phylogeographical differentiation

The SAMOVA analysis revealed that the differentiation among groups (*F*_CT_) of *N. tangutorum* reached a plateau when the value of *K* was 6 (**Supplementary Tables S2**, **Supplementary S3**; **Supplementary Figure S2**), and all populations sampled for this plant clustered into six groups. The group 6 included most of these populations (25 populations) which occurred everywhere in the whole distribution area of this species, while the group 5 consisted of 7 populations (22, 24, 26, 27, and 29-31) which occupied almost the same geographical region (**Figure 1A**). However, all the other groups (group 1-4) only included one or two populations. For the species *N. sphaerocarpa*, the SAMOVA divided all sampled populations into five groups (*K* = 5) (**Supplementary Tables S2**, **Supplementary S3**; **Supplementary Figure S2**). Except for groups 4 and 5 which contained most populations of the species (10 populations), the other groups only consisted of one or two populations. The populations of group 4 or 5 also occupied approximately the same geographical region (**Figure 1A**). The group structures based on the SAMOVA analysis were not congruent with geographical distributions of all populations of the two species. However, most populations in each close geographical region obviously contained homologous or unique chlorotype, for example, populations 33-38 in the eastern region of sampled area (Inner Mongolia) fixed unique H10, and populations 42-44 in North Xinjiang region fixed unique H15 (**Figure 1A**). Especially, almost all the populations of *N. sphaerocarpa* in the western range of Gansu province fixed the same chlorotype (H19).

To further reveal genetic structure of these populations both in the whole range and in different geographical regions, therefore, all the populations of *N. tangutorum* in this study were divided into six geographical groups (Gg1-Gg6) according to different geographical regions and climatic habitats (**Table 2**). Gg1 consisted of populations 1-7 which mainly occurred in the Qinhai-Tibet Plateau (QTP) with an average altitude of more than 4000m. Gg2 included populations 9, 12, 15, 18, and 20, which were distributed in the west of Gansu province where belongs to extreme arid area with little rainfall of less than 100 mm. The range of Gg3 (22-24, 26, 27, and 29-31) lied in arid area where the rainfall is more than 100mm but less than 200mm. Gg4 populations (33-38) are largely distributed near the Yellow River where belongs to semi-arid regions. Gg5 and Gg6 populations mainly occurred in the northern and western regions of Xinjiang, respectively, which are geographically far from the populations of other geographical groups. However, the populations of *N. sphaerocarpa* were not divided according to geographical regions, because most populations of the species in a close geographical rang (in the west of Gansu province) fixed a single chlorotype and other geographical regions contained only a few sampled populations.

**Table 2:** Estimates of average gene diversity within populations (*H*_S_), total gene diversity (*H*_T_), interpopulation differentiation (*G*_ST_), and the number of substitution types (*N*_ST_) (mean ± SE in parentheses) for chlorotypes calculated with PERMUT, using a permutation test with 1000 permutations.

Species/Geographical region	*H*_S_	*H*_T_	*G*_ST_	*N*_ST_
*N. tangutorum*	0.249 (0.040)	0.654 (0.059)	0.620 (0.059)	0.624 (0.076)^ns^
Gg1 (1-7)	0.352 (0.097)	0.818 (0.043)	0.570 (0.134)	0.662 (0.156)*
Gg2 (9,12,15,18,20)	0.142 (0.087)	0.156 (0.087)	0.092 (NC)	0.095 (NC)^ns^
Gg3 (22-24,26,27,29-31)	0.115 (0.066)	0.365 (0.173)	0.684 (NC)	0.678 (NC)^ns^
Gg4 (33-38)	0.219 (0.104)	0.257 (0.111)	0.149 (NC)	0.136 (NC)^ns^
Gg5 (39-44)	0.372 (0.100)	0.603 (0.123)	0.383 (NC)	0.373 (0.035)^ns^
Gg6 (46,48-52)	0.302 (0.119)	0.400 (0.145)	0.245 (NC)	0.211 (NC)^ns^
*N. sphaerocarpa*	0.099 (0.059)	0.758 (0.084)	0.870 (0.073)	0.932 (0.053)*

Gg, geographical group; *indicates that *N*_ST_ is significantly different from *G*_ST_ (P < 0.01); ns, not significantly different; NC, not computed due to small sample size.

**Figure 2:**
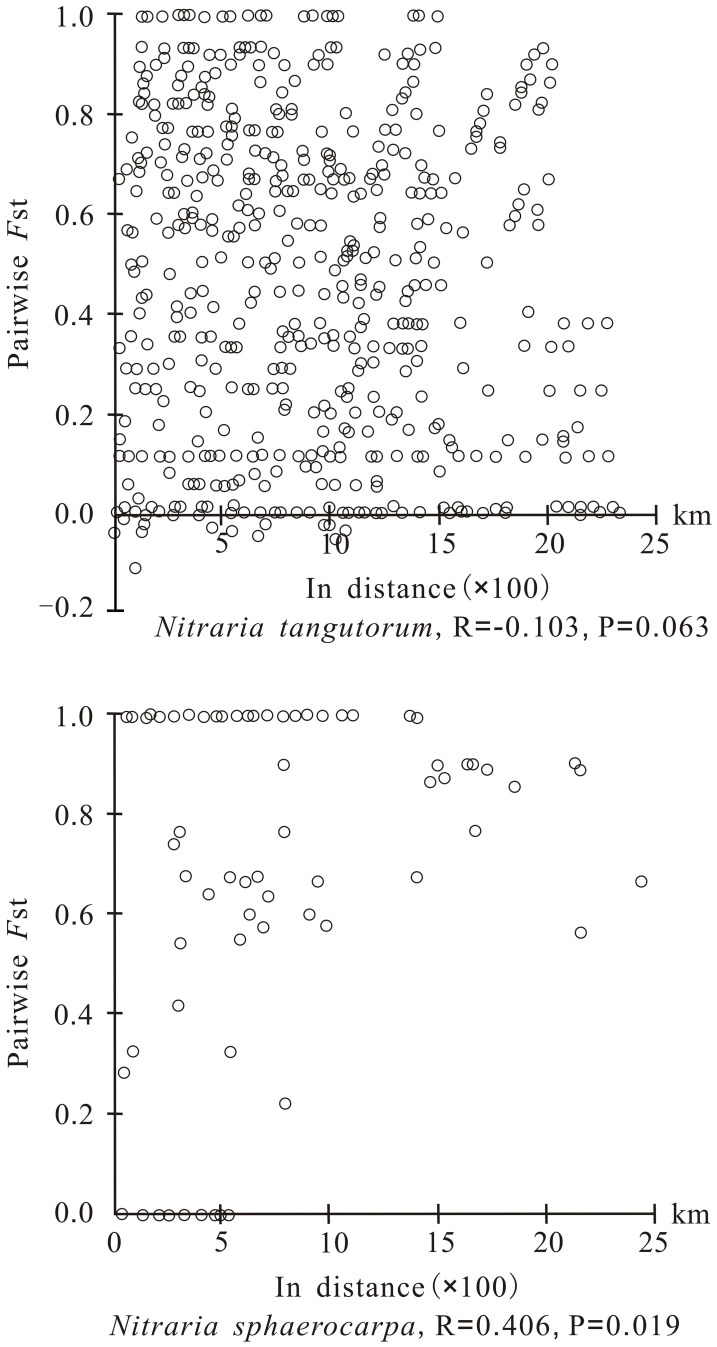
Analysis of isolation by distance for chloroplast DNA of *Nitraria tangutorum* and *Nitraria sphaerocarpa* based on Mantel test. The pairwise *F*_ST_ value is plotted against the geographical distance between populations.

The PERMUT analysis indicated that the two species, *N. tangutorum* and *N. sphaerocarpa*, have low average gene diversities within populations (*H*_S_ = 0.249 and 0.099, respectively), while the total gene diversities across all populations were high (*H*_T_ = 0.654 and 0.758, respectively) (**Table 2**). The two species also showed high level of differentiation among all the populations (*G*_ST_ = 0.620 and 0.870, *N*_ST_ = 0.624 and 0.932, respectively). The total gene diversity (*H*_T_) and interpopulation differentiation (*G*_ST_ and *N*_ST_) of *N. sphaerocarpa* were obviously higher than that of *N. tangutorum*. A permutation test detected a significant phylogeographic structure (*N*_ST_ > *G*_ST_; P < 0.01) among populations of *N. sphaerocarpa* (**Table 2**), while no clear phylogeographic structure was detected among populations of *N. tangutorum*. For the six geographical groups of *N. tangutorum* (**Table 2**: Gg1-Gg6), the total gene diversity of Gg1 in QTP (*H*_T_ = 0.818) was apparently higher than that of the other geographical groups, and only this geographical group showed a clear phylogeographic structure. The Mantel test also identified an obvious correlation between genetic distance and geographical distance for *N. tangutorum* (R = - 0.103, P = 0.063), while the correlation between the two distance matrixes was not clear for *N. sphaerocarpa* (R = 0.406, P = 0.019) (**Figure 2**).

AMOVA analyses showed that approximately 58% of the total chloroplast DNA variations occurred among populations of *N. tangutorum* whereas about 87% of variation occurred among populations of *N. sphaerocarpa*, also indicating that the level of genetic differentiation among populations of *N. sphaerocarpa* was higher than that of *N. tangutorum* (**Table 3**). For *N. tangutorum* and its six geographical groups, about 33% of variation occurred among groups (*F*_CT_ = 0.3287), revealing that there was a significant differentiation among these geographical groups. Actually, the BARRIER analysis also detected a few potential biogeographical boundaries among the populations of the two species. Two robust boundaries with high bootstrap values (> 95%) were identified among populations of *N. tangutorum* and *N. sphaerocarpa*, respectively (**Figures 1C, D**). AMOVA analysis also revealed that the molecular variation within populations of the four geographical groups (Gg2, 93.98%; Gg4, 85.76%; Gg5, 64.90%; and Gg6, 76.61%) were notably higher than that of among population within groups of *N. tangutorum* (**Table 3**).

**Table 3:** Analyses of molecular variance (AMOVA) for populations and population groups of *Nitraria tangutorum* and *Nitraria sphaerocarpa*.

Species/Geographical region	Source of variation	*df*	SS	VC	Variation (%)	Fixation index
*N. tangutorum*	Among populations	37	107.077	0.2038	57.75	*F*_ST_ = 0.577*
	Within populations	476	70.975	0.1491	42.25	
*N. sphaerocarpa*	Among populations	13	38.794	0.3464	86.87	*F*_ST_ = 0.8687*
	Within populations	105	5.500	0.0524	13.13	
*N. tangutorum* (Whole distribution)	Among groups	5	60.107	0.1220	32.87	*F*_CT_ = 0.3287*
	Among populations within groups	32	46.971	0.1000	26.95	*F*_SC_ = 0.4015*
	Within populations	476	70.975	0.1491	40.18	*F*_ST_ = 0.5982*
Gg1 (1-7)	Among populations	6	21.353	0.2092	51.10	*F*_ST_ = 0.5110*
	Within populations	108	21.621	0.2002	48.90	
Gg2 (9,12,15,18,20)	Among populations	4	0.633	0.0059	6.11	*F*_ST_ = 0.0611
	Within populations	55	4.950	0.090	93.89	
Gg3 (22-24,26,27,29-31)	Among populations	7	9.605	0.1013	68.09	*F*_ST_ = 0.6809*
	Within populations	98	4.650	0.0474	31.91	
Gg4 (33-38)	Among populations	5	2.014	0.0231	14.24	*F*_ST_ = 0.1424
	Within populations	64	8.900	0.1391	85.76	
Gg5 (39-44)	Among populations	5	8.353	0.1086	35.10	*F*_ST_ = 0.3510*
	Within populations	77	15.454	0.2007	64.90	
Gg6 (46,48-52)	Among populations	5	5.012	0.0636	23.39	*F*_ST_ = 0.2339*
	Within populations	74	15.400	0.2081	76.61	

*df*, degrees of freedom; SS, sum of squares; VC, variance components; *F*_ST_, variance among populations; *F*_SC_, variance among populations within groups; *F*_CT_, variance among groups relative to total variance. *P < 0.001.

### 3.3 Demographic history of *N. tangutorum* and *N. sphaerocarpa*

**Figure 3:**
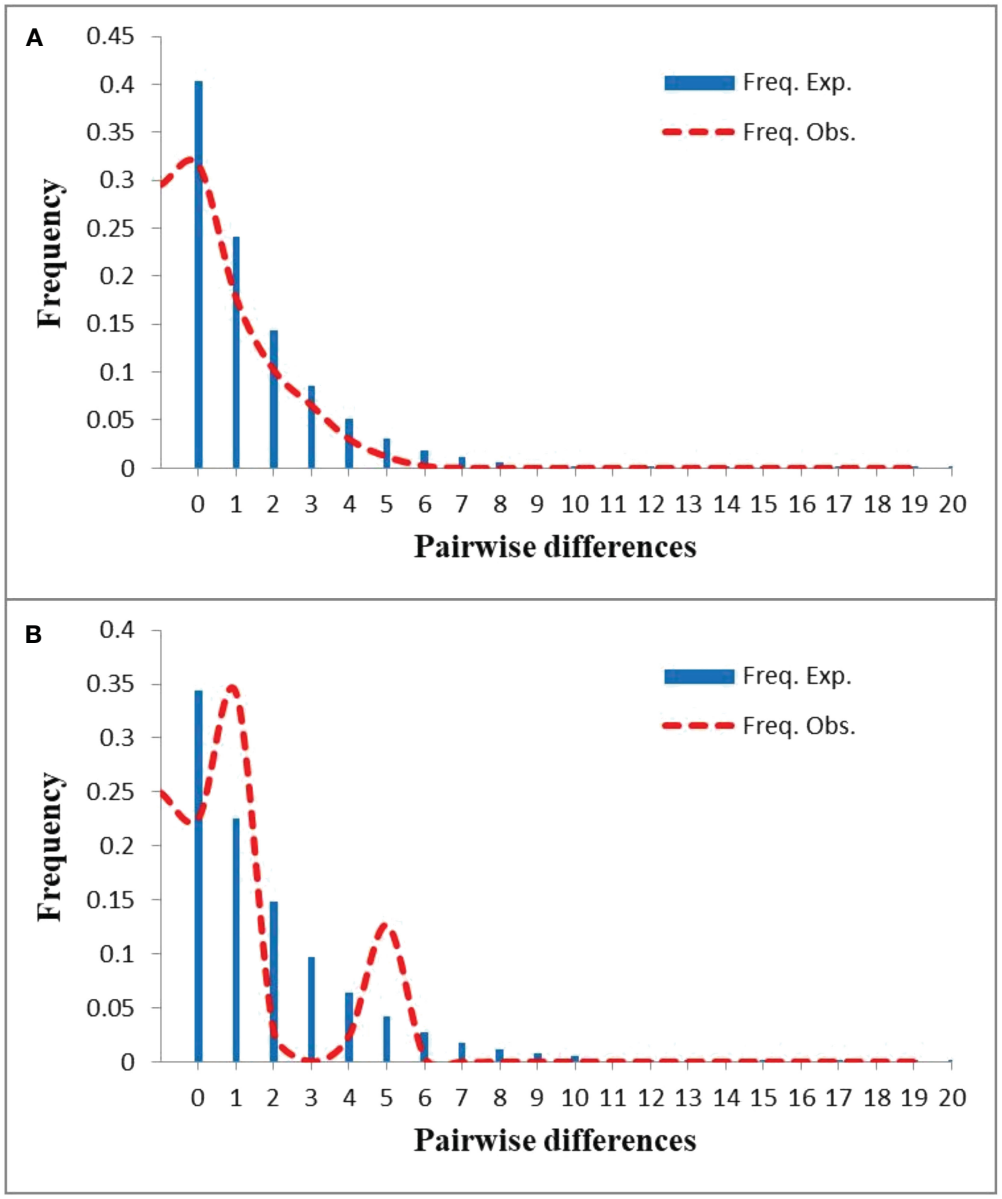
Mismatch distribution analyses for the whole distribution of *Nitraria tangutorum* **(A)** and *Nitraria sphaerocarpa* **(B)**.

The mismatch distribution analysis using DnaSP 5.10 revealed that the distribution of pairwise differences for *N. tangutorum* populations displayed a smooth and unimodal curve (**Figure 3A**), implying this species experienced a sudden demographic expansion in the past. However, the distribution of pairwise differences of *N. sphaerocarpa* was a bimodal curve (**Figure 3B**). The higher *P*-values of Harpending’s raggedness index (*RAG*) and the sum of squared deviation (*SSD*) for *N. tangutorum* (**Table 4**) further revealed that the species experienced a rapid range expansion in its whole geographical distribution. Furthermore, the obviously negative values of Tajima’s *D* and Fu’s *F*s in the whole populations of *N. tangutorum* also supported the sudden demographic expansion of the species (**Table 4**). The demographic expansion of *N. tangutorum* approximately happened between 7 and 21 Kya, according to the average mutation rates of cpDNA sequences in angiosperms (Wolfe et al., 1987), the two chloroplast DNA sequences length of 1794bp, and the generation time of 3 years. However, a rapid demographic expansion was not identified in the whole geographical distribution of the species *N. sphaerocarpa* according to statistics for neutrality tests and mismatch distribution analysis (**Table 4**).

**Table 4:** Statistics for neutrality tests and mismatch distribution analysis for the two species *Nitraria sphaerocarpa* and *Nitraria tangutorum*.

Group	τ	*SSD* (*P*-value)	*RAG* (*P*-value)	Tajima’s *D* (*P*-value)	Fu’s *F*s (*P*-value)	*t* (Kya)
*N. tangutorum*	0.462	0.0010 (0.8300)	0.0244 (0.9500)	-1.3026 (0.0876)	-5.5773 (0.0680)	7.15-21.46
*N. sphaerocarpa*	1.688	0.0471 (0.0000)	0.1395 (0.0000)	-0.2105 (0.4399)	0.8639 (0.7180)	26.14-78.41

Parameters were estimated under the sudden expansion model.

τ, time in number of generations elapsed since the sudden expansion episode; *SSD*, sum of squared deviations; *RAG*, Harpending’s raggedness index; *t*, expansion time.

**Figure 4:**
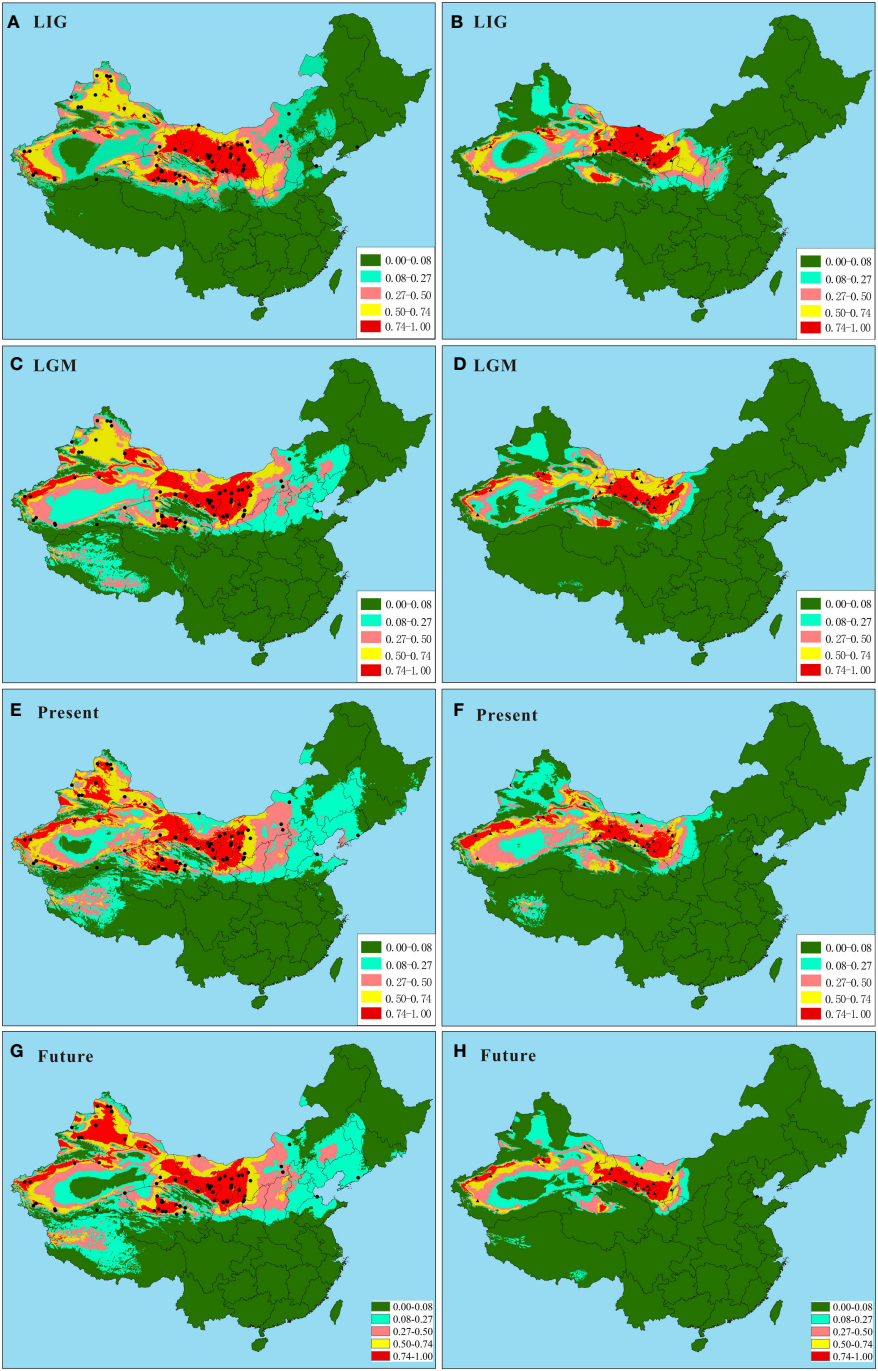
Predicted ranges of *Nitraria tangutorum* and *Nitraria sphaerocarpa* during the LIG, the LGM, the present day, and the future based on ecological niche modeling. **(A, C, E, G)** Predicted ranges of *N. tangutorum* during the LIG, the LGM, the present day, and the future, respectively. **(B, D, F, H)** Predicted ranges of *N. sphaerocarpa* during the LIG, the LGM, the present day and the future, respectively. The black plots represent the 128 sites (84 for *N. tangutorum* and 44 for *N. sphaerocarpa*), including 52 our own sampling sites and 76 specimen records from the Chinese Virtual Herbarium.

The distribution models of the two species in ENM obtained the high average AUC scores (0.875 and 0.953 for *N. tangutorum* and *N. sphaerocarpa*, respectively), based on the 30 replicates of the MAXENT runs (**Supplementary Figure S3**). Estimates of relative contributions of the environmental variables according to the jackknife tests showed that seven environmental variables, including Bio1, Bio4, Bio11, Bio12, Bio15, Bio17, and Bio18, principally influenced the geographical ranges of *N. tangutorum* and *N. sphaerocarpa* (**Supplementary Table S4**). However, contribution rates of these seven environmental factors to the distribution ranges were slightly different between the two species. Among these environmental variables used in the study, four variables, i.e. Bio1, Bio4, Bio17, and Bio18, played a primary role in determining the potential ranges of the two species, and their total contribution rate accounted for over 85% (**Supplementary Table S4**). Based on the 128 sites dataset and the seven environmental factors above, the potential distribution ranges of the two species were modeled for the LIG, the LGM, the present day, and the future, respectively (**Figures 4A–F**). The results showed that the potential ranges of the two species on the basis of a high habitat suitability index (>0.50) obviously fluctuated during the LIG and LGM (**Figures 4A–D**; **Supplementary Table S5**), comparing to the present and future ranges (**Figures 4E, F**). Especially, their potential ranges contracted significantly during the LGM period (**Figures 4C, D**; **Supplementary Table S5**), and subsequently, expanded or recolonized during the present day (**Figures 4E, F**; **Supplementary Table S5**). Furthermore, the two species will experience different degrees of range contraction under the future climate scenario, and the range contraction of *N. sphaerocarpa* is significantly greater than that of *N. tangutorum* based on a higher habitat suitability index (>0.74) (**Figures 4G, H**; **Supplementary Table S5**). Notably, the potential niche maps based on the ENM showed that the two species occupied distinct fragmented habitats in different periods, implying that the two species had multiple geographically isolated refugia in northern China. In addition, *N. tangutorum* occupied a significantly broader potential distribution range than *N. sphaerocarpa*, indicating that *N. tangutorum* had a higher ecological plasticity or adaptability to different habitats than *N. sphaerocarpa*.

## 4 Discussion

### 4.1 Genetic diversity of chloroplast DNA

In the present study, we examined high total haplotype diversity (*H*_d_ = 0.7819) and nucleotide diversity (Pi=0.00094) of the two cpDNA sequences (*trn*H-*psb*A and *atp*H-*atp*I). A total of twenty four chlorotypes were identified from 52 populations of *N. tangutorum* and *N. sphaerocarpa*. Out of these haplotypes, there was a single dominant haplotype (H4) which widely occurred in the entire geographical distribution of the two species (**Figures 1A, B**), and a lot of rare haplotypes which were fixed by only one or two individuals of them (**Table 1**; **Figure 1A**). However, the bootstrap values of most clads in the phylogenetic tree were very low (<50%, **Supplementary Figure S1**), indicating that the relationships among these chlorotypes were not clear. The PERMUT analysis indicated that the two species had high levels of total genetic diversity (**Table 2**: *N. tangutorum*: *H*_T_ = 0.654; *N. sphaerocarpa*: *H*_T_ = 0.758). In addition, we also found that four *N. tangutorum* populations (4, 34, 42, and 50) and one *N. sphaerocarpa* population (21) had obviously higher haplotype diversities than other populations (**Table 1**; **Figure 1A**), and these populations scattered different geographical regions. For example, population 4 occurs on the QTP with a series of mountains and valleys, population 42 lies in northern Xinjiang, while population 34 is distributed in central Inner Mongolia. Notably, these populations with higher haplotype diversities are geographically far from each other, and occupy different geographical regions. These results together imply that the ancestor populations of the two species were widely distributed in northwestern China before the Quaternary (Hewitt, 2000). These ancient populations have been gradually isolated into different geographical or ecological groups owing to the habitat fragmentation and formation of geographical barriers caused by past geological events and/or climate oscillations (Abbott et al., 2000; Hewitt, 2004), and subsequently formed the current genetic diversity pattern (Li et al., 2018; Jia and Zhang, 2019; Hu et al., 2022). The aridification and desert expansion in northwestern China induced by uplifting of the QTP (Sun et al., 1998; Guo et al., 2002; Sun and Liu, 2006) probably accelerated the range fragmentation, geographical subdivision and diversification of these desert species in this region (Li et al., 2012; Meng et al., 2015; Zhang et al., 2017; Hu et al., 2022). The rare haplotypes identified in this study were highly likely to be randomly retained in fragmented populations due to genetic drift during the population contraction/expansion process of the two species (e.g. Avise, 2004; Chen et al., 2008).

Although the total gene diversity of *N. sphaerocarpa* was significantly higher than that of *N. tangutorum*, most populations of *N. sphaerocarpa* were only fixed for a single haplotype (e.g. populations 8, 10, 11, 16, 17, 19, 25; **Figure 1A**). The higher *G*_ST_ value of *P. sphaerocarpa* than *N. tangutorum* indicated that the genetic differentiation among populations of *N. sphaerocarpa* was more severe than that of *N. tangutorum* (**Table 2**). AMOVA analysis also showed that approximately 87% of the total genetic variations occurred among populations of *N. sphaerocarpa*, while only about 57% variations occurred among populations of *N. tangutorum* (**Table 3**). This different genetic structure between the two related species with co-distributed ranges was probably triggered by gene flow between populations (e.g. Zink et al., 2001; Polihronakis and Caterino, 2010; Zhang et al., 2012). Habitat dependence/preference of species with similar dispersal ability probably affects the gene flow among their populations. Species with high ecological plasticity and wide ranges have more opportunities to survive and spread in adverse conditions than other species with narrow niches and small ranges (Michaux et al., 2005; Hamer and McDonnell, 2010; Lange et al., 2010; Morris-Pocock et al., 2010). *N. tangutorum* is widely distributed across large parts of northern China, covering almost all desert areas in northwestern China. Its habitats include shifting or semi-fixed sandy land, and gravel or salinized sandy land. However, *N. sphaerocarpa* is scattered in desert areas discontinuously, and only occupies some narrow desert regions in northwestern China, including Midwest of Inner Mongolia, Hexi corridor of Gansu province, and a few areas of Xinjiang. This specie prefers to grow on fixed or gravel sandy land. This habitat preferences and habitat fragmentation probably affected the gene flow between populations of *N. sphaerocarpa*, and subsequently further contribute to the genetic divergence of the species.

Notably, among the twenty-four chlorotypes detected in this study, only two chlorotypes (H4 and H14) were shared by *N. tangutorum* and *N. sphaerocarpa* (**Figure 1B**). According to species-specific chlorotype composition of two populations in the same site (e.g. population pairs: 11 and 12, 18 and 19), we inferred that the two related plants were reproductively isolated by some biological mechanisms. Therefore, these shared chlorotypes between the two species were probably derived from retention of ancestor polymorphisms, but not from hybridization or introgression. These retained ancestor haplotypes had probably experienced an incomplete lineage sorting before a complete reproductive barrier was established between the two species (Wendel and Doyle, 1998).

### 4.2 Population structure and regional differentiation

The climate change induced by uplift of the QTP since the Pliocene accelerated the desert formation and expansion in northwestern China (Guo et al., 2002), and further caused range fragmentation and subsequent diversification of plants in this region (Sun and Li, 2003; Al-Shehbaz et al., 2006). *Nitraria* is an ancient Tertiray relic taxon, and its ancestor populations were highly likely to widely occur in northern China before these geological events (Temirbayeva and Zhang, 2015; Zhang et al., 2015). Our results revealed that the two species, *N. tangutorum* and *N. sphaerocarpa*, probably experienced habitat fragmentation caused by geological and climatic changes (aridification, desert formation and expansion/contraction) (Li et al., 2012; Meng et al., 2015). The hypothesis was supported by no clear phylogenic relationship among haplotypes and geographical distributions of populations with high haplotype diversity (e.g. populations 4, 21, 34, 42, 50). These populations with high diversity were likely to be the putative glacial refugia for the species (Abbott et al., 2000). We also did not detect any significant phylogeographical structure in the entire range of *N. tangutorum* (**Table 2**), implying that all these populations of the species probably originated from different ancestor populations. The Mantel test also showed that there is no significant correlation between population genetic differentiation (*F*st) and geographical distance of *N. tangutorum* in its whole distributional ranges (**Figure 1A**; 2). Therefore, it is highly likely that the climatic oscillations and associated environmental changes (e.g. desert formation and expansion/contraction) in the Quaternary accelerated the range fragmentation and population isolation, and subsequently providing chances for allopatric differentiation within species induced by selection and/or genetic drift (Meng et al., 2015). This inference has been further confirmed by a large number of rare haplotypes identified in the study. Actually, we also found some distinctive chlorotypes in different geographical regions, e.g. H15 in northern Xinjiang region, H16, H17 and H18 in western Xinjiang region, and H3 in Qinghai (QTP region) (**Figure 1A**; **Table 1**). These unique chlorotypes were also likely to result from genetic drift and randomly retained in fragmented geographical distributions.

The population groups divided by SAMOVA were not congruent with geographical subareas of the two species, for example, the group 6 of *N. tangutorum* included 25 populations which occurred everywhere in the entire ranges of this species (**Figure 1A**; **Supplementary Table S3**). However, we did find some geographically close populations which obviously contained some homologous or unique chlorotypes, such as populations 33-38 and 22-31, indicating that phylogeographical structure existed among populations in a few subareas of *N. tangutorum* (**Table 2**). In the whole ranges of *N. sphaerocarpa*, we examined the significant phylogeographical structure (*N*_ST_ > *G*_ST_, P < 0.01) and high interpopulation differentiation (*G*_ST_ = 0.870), which may be caused by limited gene flow among populations affected by habitat preference to narrow gravel sandy land. Actually, in the subarea of Hexi corridor both in Gansu and central Inner Mongolia, we discovered obvious lineage genetic differentiation among geographical populations of *N. tangutorum* and *N. sphaerocarpa* (21-38). A few genetic barriers based on Monmonier’s maximum-difference algorithm have also been detected among these populations in the subarea of the two species (**Figures 1C, D**). This genetic pattern of the two *Nitraria* species is partly congruent with the other two typical desert taxa we surveyed using cpDNA variations (Yu et al., 2013; Zhang et al., 2017). In addition, the geographical region Gg7 (populations 1-7) of *N. tangutorum* distributed in the fringe of QTP contained three distinctive chlorotypes (H1, H3, and H5) and one rare chlorotype (H2), and had the highest total gene diversity (*H*_T_ = 0.818) and interpopulation differentiation (*G*_ST_ = 0.570), compared to other geograpihical groups (**Table 2**). A significant phylogeographical structure was also identified in this subarea group. All these results implied that a series of high mountains and deep valleys in this region played an important role in accelerating the formation of regional genetic differentiation and phylogeographical structure of the species (Dutech et al., 2004; Meng et al., 2015).

### 4.3 Population contraction/expansion and multiple refugia

A few studies have revealed that recent population expansion probably results in shallow genetic divergence (Conroy and Cook, 2000; Hewitt, 2000; 2004; Polihronakis and Caterino, 2010). The results obtained by PERMUT revealed that genetic differentiation among populations in some subareas of *N. tangutorum* (e.g. Gg2, Gg4, Gg6; **Table 2**) were very low, and most of variations occurred within populations (**Table 3**), implying that these populations in the subareas probably experienced recent regional expansion in their limited geographical regions. This scenario was further supported by mismatch analysis results (**Table 4**; **Figure 3A**). For *N. sphaerocarpa* species, we did not find a rapid demographic expansion in its whole geographical distribution (**Table 4**; **Figure 3B**), which was probably related to its habitat preference to narrow gravel sandy land. However, the ENM results revealed that the two species experienced apparent expansion and contraction during the LIG and LGM, respectively (**Figures 4A–D**; **Supplementary Table S5**), and their ranges have obviously expanded again in the present (**Figures 4E, F**; **Supplementary Table S5**). The mismatch analysis based on a range of possible cpDNA mutation rates indicated that the demographic expansion of *N. tangutorum* occurred approximately between 21 and 7 Kya before present, which was partly congruent with the ages of LGM (ca. 21 - 18 Kya before present). Numerous studies revealed that the deserts in central Asia, involving northwestern China, obviously enlarged during the Quaternary glacial ages (e.g. Bush et al., 2004; Sun and Liu, 2006; Qian et al., 2016). The range expansion of *N. tangutorum* probably followed the enlargement of the deserts during the ice ages, especially the LGM. Therefore, the recolonization of the species may have occurred after the LGM (ca. 18 - 7 Kya). This inference is basically consistent with the results of ENM and mismatch analysis. In addition, the jackknife tests in ENM revealed that two environmental variables, i.e. Bio18 and Bio4, played vital roles in modeling the potential ranges of the two species (**Supplementary Table S4**), indicating that precipitation and temperature are the two key factors for the population survival and expansion of the two species. Some studies showed that anthropogenically induced climate change within the last decade is causing shifts in the distribution ranges of many species (Walther et al., 2002; Parmesan and Yohe, 2003; Parmesan, 2006), and these range shifts are likely to continue because of carbon emissions and global climate warming. Therefore, the global warming and aridification induced by human activities will also probably produce significant effects on potential distributions of *N. tangutorum* and *N. sphaerocarpa*. The ENM results in this study indicated that the suitable ranges of the two species will contract substantially in the future climate scenario (**Figures 4G, H**; **Supplementary Table S5**), when comparing to the present (**Figures 4E, F**). Moreover, the contraction degrees of distribution ranges are obviously different between the two species.

These different demographic patterns of the two species in responses to past climate changes are probably correlated with their contrasting habitat preferences and ecological plasticity (Michaux et al., 2005; Hamer and McDonnell, 2010; Lange et al., 2010; Morris-Pocock et al., 2010). In general, phenotypic plasticity can produce morphologies adapted to local conditions, and is very beneficial for the survival of plants in heterogeneous environments (Puijalon et al., 2008). Morphogenetic differences ensure resilience of species to new and changing ecological conditions (Schoelynck et al., 2015). *N. tangutorum* and *N. sphaerocarpa* had contrasting habitat preferences and different phenotypic plasticity (Pan et al., 1999). Furthermore, *N. tangutorum* had higher average gene diversity within populations (*H*_s_) than *N. sphaerocarpa* (**Table 2**), implying that the former had higher adaptability to heterogeneous environments than the latter.

Providing that the two species extensively occurred in the Northwest of China before the Quaternary, their current populations might have originated from a few separated glacial refugia following their habitat fragmentation induced by climate oscillations in the Quaternary (Chen et al., 2008; Li et al., 2012). Firstly, the range fluctuation of one species generally remains two evident genetic imprints: a wide distribution of a dominant haplotype, and multiple rare haplotypes (Hewitt, 1996, 2000; Comes and Kadereit, 1998). In the present study, we identified a single dominant haplotype (H4) which widely occurred in the entire geographical ranges of the two species (**Figures 1A, B**), and a lot of rare haplotypes which were fixed by only one or two individuals of them (**Table 1**; **Figure 1A**). Furthermore, we also found that some different geographical regions fixed a few distinctive chlorotypes, e. g. Gg1 fixed H3, Gg3 fixed H6, Gg5 fixed H15, and Gg6 fixed H16, H17 and H18, indicating that the species have experienced regional-scale range expansion/contraction in these geographical regions. Secondly, glacial refugium areas generally contained most of haplotypes and have high levels of genetic diversity (Abbott et al., 2000), while new recolonized areas usually have low haplotype diversities owing to founder effects (e.g., Petit et al., 2003; Stewart et al., 2010), and haplotypes should be decreased gradually from refugium (Hewitt, 2000; Heuertz et al., 2004). In the present study, we found four *N. tangutorum* core populations (4, 34, 42, and 50) and one *N. sphaerocarpa* core population (21) which had obviously high levels of genetic diversity (**Table 1**; **Figure 1A**), and these core populations are scattered across different geographical regions (**Figure 1A**). Moreover, we also found that other populations near these core populations had lower levels of genetic diversity and a few chlorotypes originated from their core populations. This genetic structure implies that the two species are highly likely to have independent refugia in these separated geographical regions, and they have experienced regional demographical expansion within different geographical regions.

Topographically heterogeneous areas are likely to act as refugia for species because they facilitate survival during regional climatic stress due to availability of a range of microenvironments (Byrne et al., 2022). Generally, mountainous regions can provide a few microhabitats for species survival when they facing adverse conditions or environments, while flat regions with few major geomorphologic features usually have a poorer refugial capacity (Trew and Maclean, 2021). Therefore, topographically complex regions usually retained higher levels of genetic diversity (Garrick, 2011; Byrne et al., 2017). In the present study, the four putative refugia (populations 4, 34, 42, 50) for *N. tangutorum* had high haplotype diversities, and *H*_E_ ranged from 0.600 to 0.758 (**Table 1**). Apart from population 34 with a relatively lower level of haplotype diversity (0.600), the other three populations harbored diversity values with no significant difference among them, implying that these putative refugia had similar ecological capacities for preserving genetic diversity of the species. Furthermore, these populations with high haplotype diversities (putative refugia) are all located near some mountains (**Figure 1A**), for example, populations 34, 42 and 50, are located near the Helan Mountain, Altai Mountains, and Kunlun Mountains, respectively, although the species prefers to grow on flat sandy land in deserts.

Our results together suggest that these current populations of *N. tangutorum* and *N. sphaerocarpa* originated from a few separated glacial refugia following their habitat fragmentation in the Quarternary, and these populations have experienced regional range expansion after the LGM. Interestingly, we also found that these putative refugia in the study are mostly located near some rivers. For example, population 21 is located near the Shiyang river originating from the Qilian Mountain, 34 is located near the Yellow River, 42 is located near the Ertiz river originating from the Altai Mountains, and 50 is located near the Yarkant river originating from Kunlun Mountain. The jackknife test of the ENM revealed that Precipitation of Warmest Quarter (Bio18) is the most important factor which influenced the geographical ranges of the two species (**Supplementary Table S4**). Therefore, these seasonal or permanent rivers probably provided the possibility for the survival of the two species in different refugia when the glacial climate became dry and cold (Xu et al., 2010; Ge et al., 2011; Meng and Zhang, 2013; Meng et al., 2015).

In the present study, we only used two maternally inherited chloroplast DNA fragments to highlight the demographic history and genetic structure of closely related species with overlapping ranges in response to past geological events and climatic oscillations. Our results together suggest that the two typical desert species, *N. tanguotorum* and *N. sphaerocarpa*, responded to the past climate fluctuations in different ways due to special habitat preferences. However, phylogenetic relationships among populations in the whole geographical distribution have not been well resolved. Intraspecific gene flow among and within sub-geographical regions are not clear. Further studies using more plastid DNA fragments and nuclear genes (e. g. unlinked low-copy nuclear genes) are needed to reveal the demographic history of these desert plants with overlapping ranges.

## Data availability statement

The datasets presented in this study can be found in online repositories. The names of the repository/repositories and accession number(s) can be found below: GenBank (https://www.ncbi.nlm.nih.gov/, OR801339-OR801365).

## Author contributions

QY: Conceptualization, Formal analysis, Investigation, Methodology, Project administration, Resources, Supervision, Writing – original draft. JH: Methodology, Resources, Writing – review & editing. XH: Methodology, Resources, Writing – review & editing. YZ: Supervision, Writing – review & editing. FW: Resources, Writing – review & editing. SJ: Methodology, Writing – review & editing. YW: Methodology, Writing – review & editing.

## References

[B1] Abbott R. J. Smith L. C. Milne R. I. Crawford R. M. Wolff K. Balfour J. (2000). Molecular analysis of plant migration and refugia in the Arctic. Science 289, 1343–1346. doi: 10.1126/science.289.5483.1343 10958779

[B2] Al-Shehbaz I. A. Beilstein M. A. Kellogg E. A. (2006). Systematics and phylogeny of the Brassicaceae (Cruciferae): an overview. Plant Syst. Evol. 259, 89–120. doi: 10.1007/s00606-006-0415-z

[B3] Avise J. C. (2004). Molecular markers, natural history, and evolution (Sunderland: inauer Associates).

[B4] Bandelt H. J. Forster P. Röhl A. (1999). Median-joining networks for inferring intraspecific phylogenies. Mol. Biol. Evol. 16, 37–48. doi: 10.1093/oxfordjournals.molbev.a026036 10331250

[B5] Bennett K. D. (1997). Evolution and ecology: The pace of life (Cambridge: Cambridge University Press).

[B6] Bush A. B. G. Little E. C. Rokosh D. White D. Rutter N. W. (2004). Investigation of the spatio-temporal variability in Eurasian Late Quaternary loess-paleosol sequences using a coupled atmosphere-ocean general circulation model. Quaternary Sci. Rev. 23, 481–498. doi: 10.1016/j.quascirev.2003.08.009

[B7] Byrne M. Millar M. A. Coates D. Macdonald B. M. McArthur S. M. Zhou M.. (2017). Phylogeographic evidence for inland ranges being mesic refugia for a widespread eucalypt in an arid landscape. J. Biogeogr. 44, 2539–2550. doi: 10.1111/jbi.13057

[B8] Byrne M. Ramalho C. E. Tapper S. Coates D. J. (2022). Topographic complexity facilitates persistence compared to signals of contraction and expansion in the adjacent subdued landscape. Font. Conserv. Sci. 3. doi: 10.3389/fcosc.2022.833766

[B9] Cain S. A. (1994). Foundation of plant geography (New York: Harper and Row).

[B10] Chen K. M. Abbott R. J. Milne R. I. Tian X. M. Liu J. Q. (2008). Phylogeography of *Pinus tabulaeformis* Carr. (Pinaceae), a dominant species of coniferous forest in northern China. Mol. Ecol. 17, 4276–4288. doi: 10.1111/j.1365-294X.2008.03911.x 19378405

[B11] Comes H. P. Kadereit J. W. (1998). The effect of Quaternary climatic changes on plant distribution and evolution. Trends Plant Sci. 3, 432–438. doi: 10.1016/S1360-1385(98)01327-2

[B12] Conroy C. J. Cook J. A. (2000). Phylogeography of a post-glacial colonizer: Microtus longicaudus (Rodentia: muridae). Mol. Ecol. 9, 165–175. doi: 10.1046/j.1365-294x.2000.00846.x 10672160

[B13] Dong W. Liu. J. Yu. J. Wang L. Zhou S. (2012). Highly variable chloroplast markers of evaluating plant phylogeny at low taxonomic levels and for DNA barcoding. PLoS One 7, e35071. doi: 10.1371/journal.pone.0035071 22511980 PMC3325284

[B14] Dupanloup I. Schneider S. Excoffier L. (2002). A simulated annealing approach to define the genetic structure of populations. Mol. Ecol. 11, 2571–2581. doi: 10.1046/j.1365-294X.2002.10650.x 12453240

[B15] Dutech C. Joly H. I. Jarne P. (2004). Gene flow, historical population dynamics and genetic diversity within French Guianan populations of a rainforest tree species, *Vouacapoua americana* . Heredity 92, 69–77. doi: 10.1038/sj.hdy.6800384 14679389

[B16] Excoffier L. Laval G. Schneider S. (2005). Arlequin (version 3.0): an integrated software package for population genetics data analysis. Evol. Bioinform. 1, 47–50. doi: 10.1143/JJAP.34.L418 PMC2658868 19325852

[B17] Fawcett T. (2006). An introduction to ROC analysis. Pattern Recogn. Lett. 27, 861–874. doi: 10.1016/j.patrec.2005.10.010

[B18] Felsenstein J. (1985). Confidence limits on phylogenies: an approach using the bootstrap. Evolution 39, 783–791. doi: 10.2307/2408678 28561359

[B19] Felsenstein J. (2005). PHYLIP (Phylogeny Inference Package) version 3.6. Distributed by the author (Seattle: Department of Genome Sciences, University of Washington).

[B20] Fu Y. X. (1997). Statistical tests of neutrality of mutations against population growth, hitchhiking and background selection. Genetics 147, 915–925. doi: 10.1093/genetics/147.2.915 9335623 PMC1208208

[B21] Garrick R. (2011). Montane refuges and topographic complexity generate and maintain invertebrate biodiversity: recurring themes across space and time. J. Insect Conserv. 15, 469–478. doi: 10.1007/s10841-010-9349-4

[B22] Ge X. J. Hwang C. C. Liu Z. H. Huang C. C. Huang W. H. Hung K. H.. (2011). Conservation genetics and phylogeography of endangered and endemic shrub *Tetraena mongolica* (Zygophyllaceae) in Inner Mongolia, China. BMC Genet. 12, 1. doi: 10.1186/1471-2156-12-1 21205287 PMC3025899

[B23] Guo Z. T. Ruddiman W. F. Hao Q. Z. Wu H. B. Qiao Y. S. Zhu R. X.. (2002). Onset of Asian desertification by 22 Myr ago inferred from loess deposits in China. Nature 416, 159–163. doi: 10.1038/416159a 11894089

[B24] Hamer A. J. McDonnell M. J. (2010). The response of herpetofauna to urbanization: inferring patterns of persistence from wildlife databases. Aust. Ecol. 35, 568–580. doi: 10.1111/j.1442-9993.2009.02068.x

[B25] Haring E. Gamauf A. Kryukov A. (2007). Phylogeographic patterns in widespread corvid birds. Mol. Phylogenet. Evol. 45, 840–862. doi: 10.1016/j.ympev.2007.06.016 17920300

[B26] Harpending H. C. (1994). Signature of ancient population growth in a low-resolution mitochondrial DNA mismatch distribution. Hum. Biol. 66, 591–600. doi: 10.1038/hdy.1994.122 8088750

[B27] Heuertz M. Fineschi S. Anzidei M. (2004). Chloroplast DNA variation and postglacial recolonization of common ash (*Fraxinus excelsior* L.) in Europe. Mol. Ecol. 13, 3437–3452. doi: 10.1111/j.1365-294X.2004.02333.x 15488002

[B28] Hewitt G. M. (1996). Some genetic consequences of ice ages, and their role in divergence and speciation. Biol. J. Linn. Soc 58, 247–276. doi: 10.11111/j.1095-8312.1996.tb01434

[B29] Hewitt G. M. (2000). The genetic legacy of the Quaternary ice ages. Nature 405, 907–913. doi: 10.1038/35016000 10879524

[B30] Hewitt G. M. (2004). Genetic consequences of climatic oscillations in the Quaternary. Philosophical Transactions of the Royal Society of London. Ser. B Biol. Sci. 359, 183–195. doi: 10.1098/rstb.2003.1388 PMC1693318 15101575

[B31] Hijmans R. J. Cameron S. E. Parra J. L. Jones P. G. Jarvis A. (2005). Very high resolution interpolated climate surfaces for global land areas. Int. J. Climatol. 25, 1965–1978. doi: 10.1002/joc.1276

[B32] Hu X. K. Hu J. Zhang Y. H. Jiang S. X. Yu Q. S. (2022). Genetic structure and demographic history of *Allium mongolicum* based on SSR markers. Plant Syst. Evol. 308, 2–15. doi: 10.1007/s00606-021-01802-y

[B33] Jia S. W. Zhang M. L. (2019). Pleistocene climate change and phylogeographic structure of the *Gymnocarpos przewalskii* (Caryophyllaceae) in the northwest China: Evidence from plastid DNA, ITS sequences and Microsatellite. Ecol. Evol. 9, 5219–5235. doi: 10.1002/ece3.5113 31110674 PMC6509395

[B34] Kirchman J. J. Franklin J. D. (2007). Comparative phylogeography and genetic structure of Vanuatu birds: control region variation in a rail, a dove, and a passerine. Mol. Phylogenet. Evol. 43, 14–23. doi: 10.1016/j.ympev.2006.12.013 17321760

[B35] Lange R. Durka W. Holzhauer S. I. J. Wolters V. Diekotter T. (2010). Differential threshold effects of habitat fragmentation on gene flow in two widespread species of bush crickets. Mol. Ecol. 19, 4936–4948. doi: 10.1111/j.1365-294x.2010.04877.x 20964760

[B36] Li Z. H. Chen J. Zhao G. F. Guo Y. P. Kou Y. X. Ma Y. Z.. (2012). Response of a desert shrub to past geological and climatic change: A phylogeographic study of *Reaumuria soongorica* (Tamaricaceae) in western China. J. Syst. Evol. 50, 351–361. doi: 10.1111/j.1759-6831.2012.00201.x

[B37] Li Y. Gao Q. B. Gengji Z. M. Jia L. K. Wang Z. H. Chen S. L. (2018). Rapid intraspecific diversification of the alpine species *Saxifraga sinomontana* (Saxifragaceae) in the Qinghai-Tibetan Plateau and Himalayas. Front. Genet. 9. doi: 10.3389/fgene.2018.00381 PMC6153349 30279701

[B38] Li Q. H. Xu J. Gao T. T. Yan X. Xin Z. M. (2013). Breeding system characteristics, blooming and fruiting habits of Nitraria L. J. Northeast Forestry Univ. 41, 68–71. doi: 10.3969/j.issn.1000-5382.2013.09.017

[B39] Li S. F. Zhang Q. C. Zhang Q. C. Zong C. W. Tian X. F. (2005). Research advance of genus *Nitraria* . J. Beihua Univ. (Natural Science) 6, 78–81. doi: 10.3969/j.issn.1009-4822.2005.01.022

[B40] Librado P. Rozas J. (2009). DnaSP v5: a software for comprehensive analysis of DNA polymorphism data. Bioinformatics 25, 1451–1452. doi: 10.1093/bioinformatics/btp187 19346325

[B41] Liu S. W. (1999). Flora Qinghaiica Vol. 1-4 (Xining: Qinghai People’s Publishing House).

[B42] Liu C. R. Berry P. M. Dawson T. P. Pearson R. G. (2010). Selecting thresholds of occurrence in the prediction of species distributions. Ecography 28, 385–393. doi: 10.1111/j.0906-7590.2005.03957.x

[B43] Liu Y. F. Wang Y. Huang H. W. (2009). Species-level phylogeographical history of *Myricaria* plants in the mountain ranges of western China and the origin of *M. laxiflora* in the three gorges mountain region. Mol. Ecol. 18, 2700–2712. doi: 10.1111/j.1365-294X.2009.04214.x 19457193

[B44] Liu L. P. Xu Z. M. He Z. (2016). Nutritional components of fruits of *Nitraria* L. and its utilization in Alashan desert area. J. Inner Mongolia Forestry Sci. Technol. 42, 29–31. doi: 10.3969/j.issn.1007-4066.2016.03.007

[B45] Manni F. Guérard E. Heyer E. (2004). Geographic patterns of (genetic, morphologic, linguistic) variation: how barriers can be detected by using Monmonier’s algorithm. Hum. Biol. 76, 173–190. doi: 10.1353/hub.2004.0034 15359530

[B46] Mantel N. (1967). The detection of disease clustering and a generalized regression approach. Cancer Res. 59, 209–220. doi: 10.1007/s00253-002-1013-9 6018555

[B47] Meng H. H. Gao X. Y. Huang J. F. Zhang M. L. (2015). Plant phylogeography in arid Northwest China: Retrospective and perspectives. J. Syst. Evol. 53, 33–46. doi: 10.1111/jse.12088

[B48] Meng H. H. Zhang M. L. (2013). Diversification of plant species in arid Northwest China: Species-level phylogeographical history of *Lagochilus* Bunge ex Bentham (Lamiaceae). Mol. Phylogenet. Evol. 68, 398–409. doi: 10.1016/j.ympev.2013.04.012 23629053

[B49] Michaux J. R. Libois R. Filippucci M. G. (2005). So close and so different: comparative phylogeography of two small mammal species, the Yellownecked fieldmouse (Apodemus flavicollis) and the Woodmouse (Apodemus sylvaticus) in the Western Palearctic region. Heredity 94, 52–63. doi: 10.1038/sj.hdy.6800561 15329664

[B50] Morris-Pocock J. A. Steeves T. E. Estela F. A. Anderson D. J. Friesen V. L. (2010). Comparative phylogeography of brown (Sula leucogaster) and red-footed boobies (S. sula): the influence of physical barriers and habitat preference on gene flow in pelagic seabirds. Mol. Phylogenet. Evol. 54, 883–896. doi: 10.1016/j.ympev.2009.11.013 19931624

[B51] Nei M. (1987). Molecular Evolutionary Genetics (New York: Columbia University Press).

[B52] Palmé A. E. Semerikov V. Lascoux M. (2003). Absence of geographical structure of chloroplast DNA variation in sallow, *Salix caprea* L. Heredity 91, 465–474. doi: 10.1038/sj.hdy.6800307 14576739

[B53] Pan X. L. Shen G. M. Chen P. (1999). A preliminary research on taxonomy and systematics of genus *Nitraria* . Acta Bot. Yunnan 21, 287–295. doi: 10.3969/j.issn.2095-0845.1999.03.002

[B54] Pan X. Y. Wei X. P. Yu Q. S. Chen J. K. Wang G. X. (2003). Polyploidy: Classification, evolution and applied perspective of the genus *Nitraria* L. Chin. Bull. Bot. 20, 632–638. doi: 10.3969/j.issn.1674-3466.2003.05.016

[B55] Parmesan C. (2006). Ecological and evolutionary response to recent climate change. Ann. Rev. Ecol. Evol. Syst. 37, 637–669. doi: 10.1146/annurev.ecolsys.37.091305.110100

[B56] Parmesan C. Yohe G. (2003). A globally coherent fingerprint of climate change impacts across natural systems. Nature 421, 37–42. doi: 10.1038/nature01286 12511946

[B57] Peterson A. T. Soberón J. (2012). Species distribution modeling and ecological niche modeling: getting the concepts right. Nat. Conservacao 10, 102–107. doi: 10.4322/natcon.2012.019

[B58] Petit R. J. Aguinagalde I. de Beaulieu J. L. Bittkau C. Brewer S. Cheddadi R.. (2003). Glacial refugia: Hotspots but not melting pots of genetic diversity. Science 300 (5625), 1563–1565. doi: 10.1126/science.1083264 12791991

[B59] Phillips S. J. Anderson R. P. Schapire R. E. (2006). Maximum entropy modeling of species geographic distributions. Ecol. Model. 190, 231–259. doi: 10.1016/j.ecolmodel.2005.03.026

[B60] Phillips S. J. Dudik M. Schapire R. E. (2004). A maximum entropy approach to species distribution modeling. In Proceedings of the 21st International Conference on Machine Learning, New York: Association for Computing Machinery Press. 655–662. doi: 10.1145/1015330.1015412

[B61] Polihronakis M. Caterino M. S. (2010). Contrasting patterns of phylogeographic relationships in sympatric sister species of ironclad beetles (Zopheridae: Phloeodes spp.) in California’s Transverse Ranges. BMC Evol. Biol. 10, 195. doi: 10.1186/1471-2148-10-195 20573263 PMC2904329

[B62] Pons O. Petit R. J. (1996). Measuring and testing genetic differentiation with ordered versus unordered alleles. Genetics 144, 1237–1245. doi: 10.1093/genetics/144.3.1237 8913764 PMC1207615

[B63] Puijalon S. Léna J. P. Rivière N. Champagne J. Y. Rostan J. C. Bornette G. (2008). Phenotypic plasticity in response to mechanical stress: hydrodynamic performance and fitness of four aquatic plant species. New Phytol. 177, 907–917. doi: 10.1111/j.1469-8137.2007.02314.x 18275493

[B64] Qian C. J. Yin H. X. Shi Y. Zhao J. C. Yin C. L. Luo W. Y.. (2016). Population dynamics of *Agriophyllum squarrosum*, a pioneer annual plant endemic to mobile sand dunes, in response to global climate change. Sci. Rep. 6, 26613. doi: 10.1038/srep26613 27210568 PMC4876407

[B65] Rogers A. R. Harpending H. (1992). Population growth makes waves in the distribution of pairwise genetic differences. Molec. Biol. Evol. 9, 552–569. doi: 10.1093/oxfordjournals.molbev.a040727 1316531

[B66] Schneider S. Excoffier L. (1999). Estimation of past demographic parameters from the distribution of pairwise differences when the mutation rates vary among sites: application to human mitochondrial DNA. Genetics 152, 1079–1089. doi: 10.1093/genetics/152.3.1079 10388826 PMC1460660

[B67] Schoelynck J. Puigalon S. Meire P. Struyf E. (2015). Thigmomorphogenetic responses of an aquatic macrophyte to hydrodynamic stress. Front. Plant Sci. 6. doi: 10.3389/fpls.2015.00043 PMC4318274 25699070

[B68] Shaw J. Lickey E. B. Schilling E. E. Small R. A. (2007). Comparison of whole chloroplast genome sequences to choose noncoding regions for phylogenetic studies in angiosperms: the tortoise and the hare III. Amer. J. Bot. 94, 275–288. doi: 10.3732/ajb.94.3.275 21636401

[B69] Shi Y. Y. Yang J. H. Zuo F. Y. Xu H. J. Liu T. Gong W. Q. (2014). Effects of salt stress on germination of three species of *Nitraria* and their salt tolerance analysis. J. Henan Agric. Sci. 43, 124–128. doi: 10.3969/j.issn.1004-3268.2014.09.027

[B70] Slatkin M. Hudson R. R. (1991). Pairwise comparisons of mitochondrial DNA sequences in stable and exponentially growing populations. Genetics 129, 555–562. doi: 10.1093/genetics/129.2.555 1743491 PMC1204643

[B71] Stewart J. R. Lister A. M. Barnes I. Dalen L. (2010). Refugia revisited: individualistic responses of species in space and time. Proc. Biol. Sci. 277, 661–671. doi: 10.1098/rspb.2009.1272 19864280 PMC2842738

[B72] Sun J. M. Ding Z. L. Liu T. S. (1998). Desert distributions during the glacial maximum and climatic optimum: Example of China. Episodes 21, 28–31. doi: 10.18814/epiiugs/1998/v21i1/005

[B73] Sun H. Li Z. M. (2003). Qinghai-Tibet Plateau uplift and its impact on tethhys flora. Adv. Earth Sci. 18, 852–862. doi: 10.3321/j.issn:1001-8166.2003.06.004

[B74] Sun J. M. Liu T. S. (2006). The age of the Taklimakan desert. Science 312, 1621. doi: 10.1126/science.1124616 16778048

[B75] Taberlet P. Fumagalli L. Wust-Saucy A. G. Cosson J. F. (1998). Comparative phylogeography and postglacial colonization routes in Europe. Mol. Ecol. 7, 453–464. doi: 10.1046/j.1365-294x.1998.00289.x 9628000

[B76] Taberlet P. Gielly L. Pautou G. Bouvet J. (1991). Universal primers for amplification of three non-coding regions of chloroplast DNA. Plant Mol. Biol. 17, 1105–1109. doi: 10.1007/BF00037152 1932684

[B77] Tajima F. (1989). Statistical method for testing the neutral mutation hypothesis by DNA polymorphism. Genetics 123, 585–595. doi: 10.1101/gad.3.11.1801 2513255 PMC1203831

[B78] Tamura K. Dudley J. Nei M. Mumar S. (2007). MEGA4: molecular evolutionary genetics analysis (GEGA) software version 4.0. Mol. Bio. Evol. 24, 1596–1599. doi: 10.1093/molbev/msm092 17488738

[B79] Temirbayeva K. Zhang M. L. (2015). Molecular phylogenetic and biogeographical analysis of *Nitraria* based on nuclear and chloroplast DNA sequences. Plant Syst. Evol. 301, 1897–1906. doi: 10.1007/s00606-015-1202-5

[B80] Thompson J. D. Gibson T. J. Plewinak F. Jeanmougin F. Higgins D. G. (1997). The Clustal-X windows interface: flexible strategies for multiple sequence alignment aided by quality analysis tools. Nucleic Acids Res. 25, 4876–4882. doi: 10.1093/NAR/25.24.4876 9396791 PMC147148

[B81] Trew B. T. Maclean M. D. (2021). Vulnerability of global biodiversity hotspots to climate change. Glob. Ecol. Biogeogr. 30, 768–783. doi: 10.1111/geb.13272

[B82] Walther G. R. Post E. Convey P. Menzel A. Parmesan C. Beebee T. J. C.. (2002). Ecological responses to recent climate change. Nature 416, 389–395. doi: 10.1038/416389a 11919621

[B83] Wan D. S. Feng J. J. Jiang D. C. Mao K. S. Duan Y. W. Miehe G.. (2016). The Quaternary evolutionary history, potential distribution dynamics, and conservation implications for a Qinghai-Tibet Plateau endemic herbaceous perennial, *Anisodus tanguticus* (Solanaceae). Ecol. Evol. 6, 1977–1995. doi: 10.1002/ece3.2019 27099706 PMC4831433

[B84] Wang Y. F. Xie Q. J. Ding X. Y. Sun X. G. Luo S. P. He K. B. (2018). Study of inhibitory action of BaiCi on gastric cancer MGC-803 cells. Western J. Traditional Chin. Med. 31, 14–16. doi: CNKI:SUN:GSZY.0.2018-05-005

[B85] Wendel J. F. Doyle J. J. (1998). “Phylogenetic incongruence: window into genome history and molecular evolution,” in Molecular Systematics of Plants II. Eds. Soltis D. E. Soltis P. S. Doyle J. J. (Springer, New York City), 265–296. doi: 10.1007/978-1-4615-5419-6_10

[B86] Wolfe K. H. Li W. H. Sharp P. M. (1987). Rates of nucleotide substitution vary greatly among plant mitochondrial, chloroplast, and nuclear DNAs. Proc. Natl. Acad. Sci. U.S.A. 84, 9054–9058. doi: 10.1073/pnas.84.24.9054 3480529 PMC299690

[B87] Wright S. (1978). Evolution and the Genetics of Populations 4. Variability Within and Among Natural Populations (Chicago, Illinois: University of Chicago Press).

[B88] Wu L. L. Cui X. K. Milne R. I. Sun Y. S. Liu J. Q. (2010). Multiple autopolyploidizations and range expansion of *Allium przewalskianum* Regel. (Alliaceae) in the Qinghai-Tibetan Plateau. Mol. Ecol. 19, 1691–1704. doi: 10.1111/j.1365-294X.2010.04613.x 20345685

[B89] Xu L. R. Huang C. J. Liu Y. X. Li B. T. Zhang Y. T. (1998). “Zygophyllaceae,” in Flora of China (43) (Science Press, Beijing), 118–122.

[B90] Xu X. Kleidon A. Miller L. Wang S. Wang L. (2010). Late Quaternary glaciation in the Tianshan and implications for palaeoclimatic change: a review. Boreas 39, 215–232. doi: 10.1111/j.1502-3885.2009.00118.x

[B91] Yang Q. Z. (2006). A discussion about new record in W. Sichuan and discontinuous distribution ways of Australia for Genus *Nitraria* L. J. Mount. Sci. 24, 137–143. doi: 10.3969/j.issn.1008-2786.2006.02.002

[B92] Yang D. Fang X. M. Dong G. R. Peng Z. C. Li J. J. (2006). Aeolian deposit evidence for formation and evolution of the Tengger Desert in the north of China since early Pleistocene. Mar. Geology Quaternary Geology 26, 93–100. doi: 10.1111/j.1745-4557.2006.00081.x

[B93] Yin H. X. Yan X. Shi Y. Qian C. J. Li Z. H. Zhang W.. (2015). The role of east Asian monsoon system in shaping population divergence and dynamics of a constructive desert shrub *Reaumuria soongarica* . Sci. Rep. 5, 15823. doi: 10.1038/srep15823 26510579 PMC4625182

[B94] Yu Q. S. Wang Q. Wu G. L. Ma Y. Z. He X. Y. Wang X.. (2013). Genetic differentiation and delimitation of *Pugionium dolabratum* and *Pugionium cornutum* (Brassicaceae). Plant Syst. Evol. 299, 1355–1365. doi: 10.1007/s00606-013-0800-3

[B95] Zhang R. Y. Song G. Qu Y. H. Alström P. Ramos R. Xing X. Y.. (2012). Comparative phylogeography of two widespread magpies: Importance of habitat preference and breeding behavior on genetic structure in China. Mol. Phylogenet. Evol. 65, 562–572. doi: 10.1016/j.ympev.2012.07.011 22842292

[B96] Zhang M. L. Temirbayeva K. Sanderson S. C. Chen X. (2015). Young dispersal of xerophil *Nitraria* lineages in intercontinental disjunctions of the Old World. Sci. Rep. 5, 13840. doi: 10.1038/srep13840 26343223 PMC4561381

[B97] Zhang Y. H. Yu Q. S. Zhang Q. Hu X. K. Hu J. Fan B. L. (2017). Regional-scale differentiation and phylogeography of a desert plant *Allium mongolicum* (Liliaceae) inferred from chloroplast DNA sequence variation. Plant Syst. Evol. 303, 451–466. doi: 10.1007/s00606-016-1383-6

[B98] Zink R. M. (1996). Comparative phylogeography in North American birds. Evolution 50, 308–317. doi: 10.2307/2410802 28568862

[B99] Zink R. M. Kessen A. E. Line T. V. Blackwell-Rago R. C. (2001). Comparative phylogeography of some aridland bird species. Condor 103, 1–10. doi: 10.1650/0010-5422(2001)103[0001:CPOSAB]2.0.CO;2

