# Supplementary material for: Demographic patterns of two related desert shrubs with overlapping distributions in response to past climate changes
Source: Front Plant Sci. 2024 Feb 21;15:1345624. doi: 10.3389/fpls.2024.1345624 (PMC10915042; doi:10.3389/fpls.2024.1345624)
Supplement: Supplementary file 1 [file Image_1.pdf]

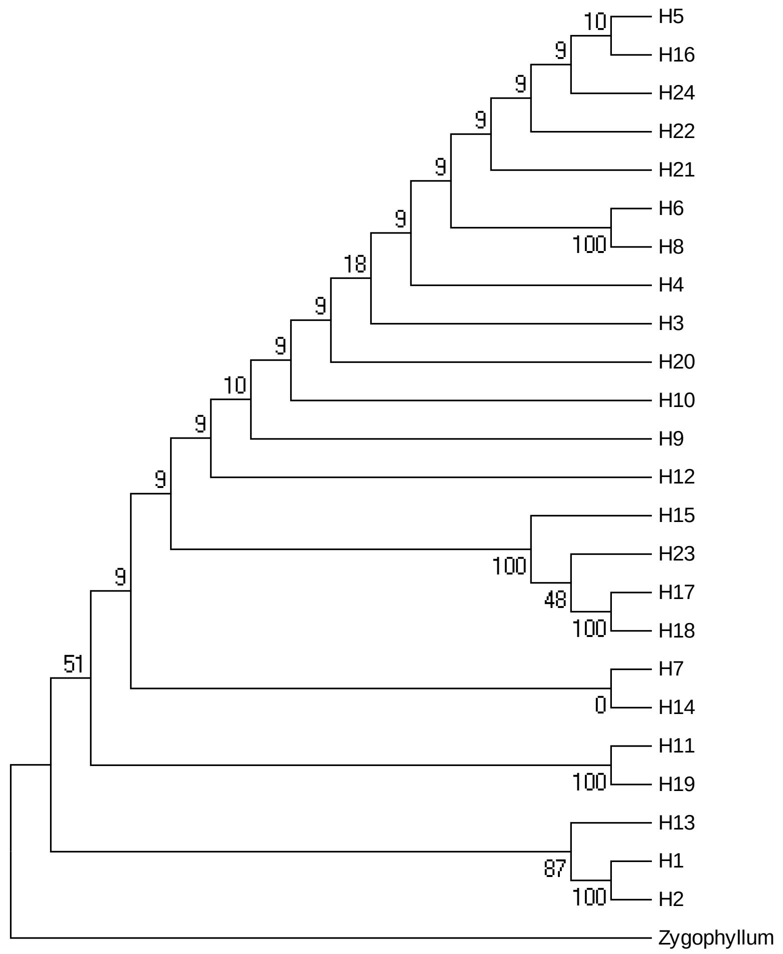

Supplementary Figure S1 Phylogenetic relationships of twenty-four chlorotypes resolved in *Nitraria. tangutorum and N. sphaerocarpa* using *Zygophyllum xanthoxylum* as outgroup. The maximum parsimony tree is presented. This has the same topology as neighbour-joining and maximum likehood trees produced from the same data.
