# Supplementary material for: Demographic patterns of two related desert shrubs with overlapping distributions in response to past climate changes
Source: Front Plant Sci. 2024 Feb 21;15:1345624. doi: 10.3389/fpls.2024.1345624 (PMC10915042; doi:10.3389/fpls.2024.1345624)
Supplement: Supplementary file 2 [file Image_2.pdf]

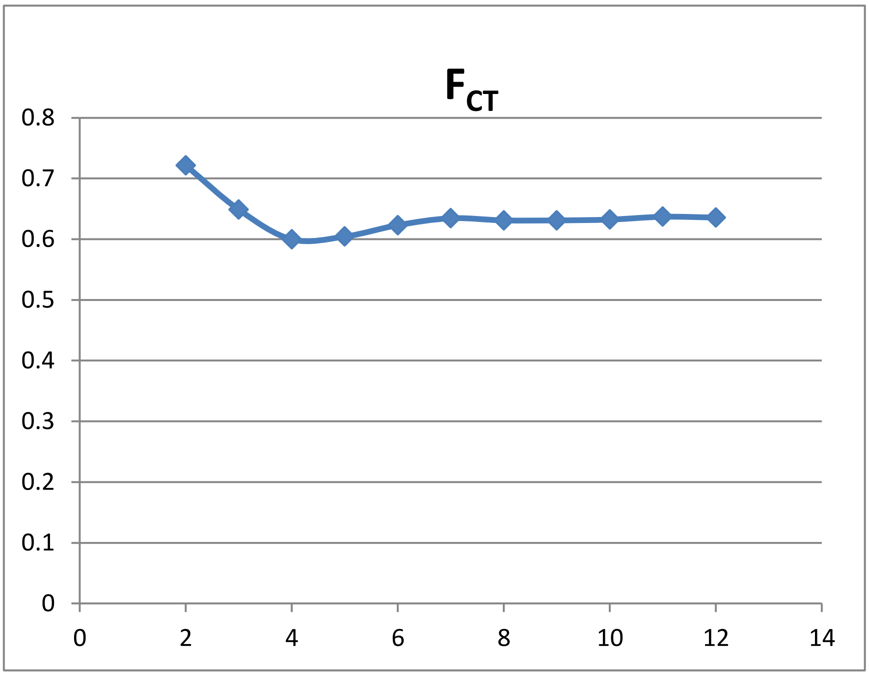

Nitraria tangutorum

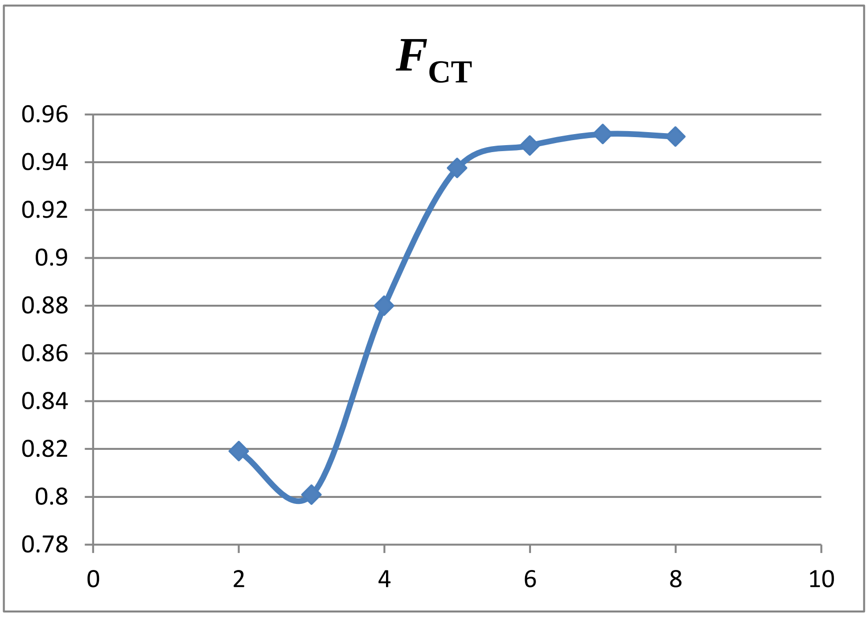

Nitraria sphaerocarpa

Supplementary Figure S2 The trend of F_{CT} value wih the number of groups (K) of *Nitraria tangutorum* and *Nitraria sphaerocarpa*.
