# Supplementary material for: Demographic patterns of two related desert shrubs with overlapping distributions in response to past climate changes
Source: Front Plant Sci. 2024 Feb 21;15:1345624. doi: 10.3389/fpls.2024.1345624 (PMC10915042; doi:10.3389/fpls.2024.1345624)
Supplement: Supplementary file 3 [file Image_3.pdf]

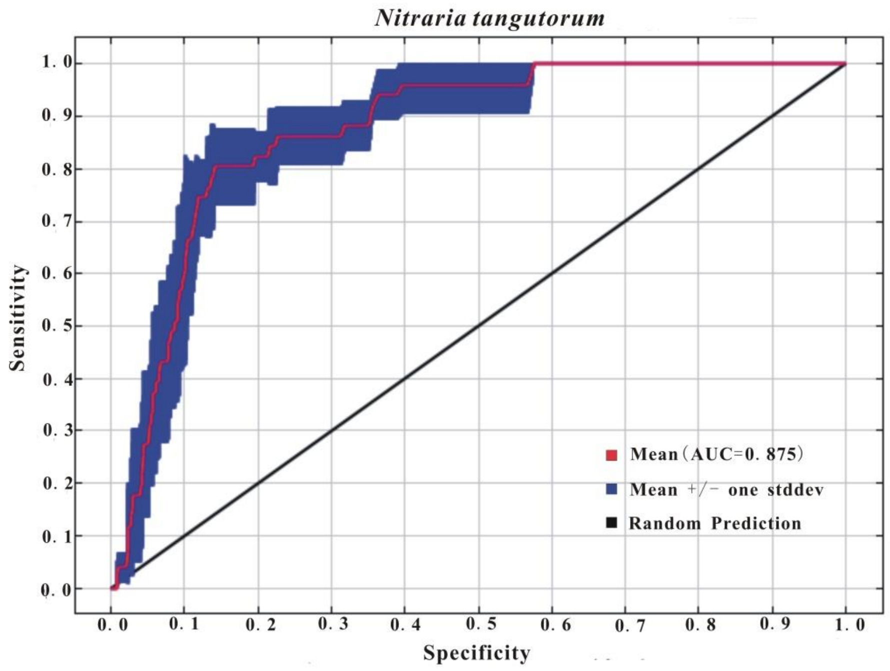

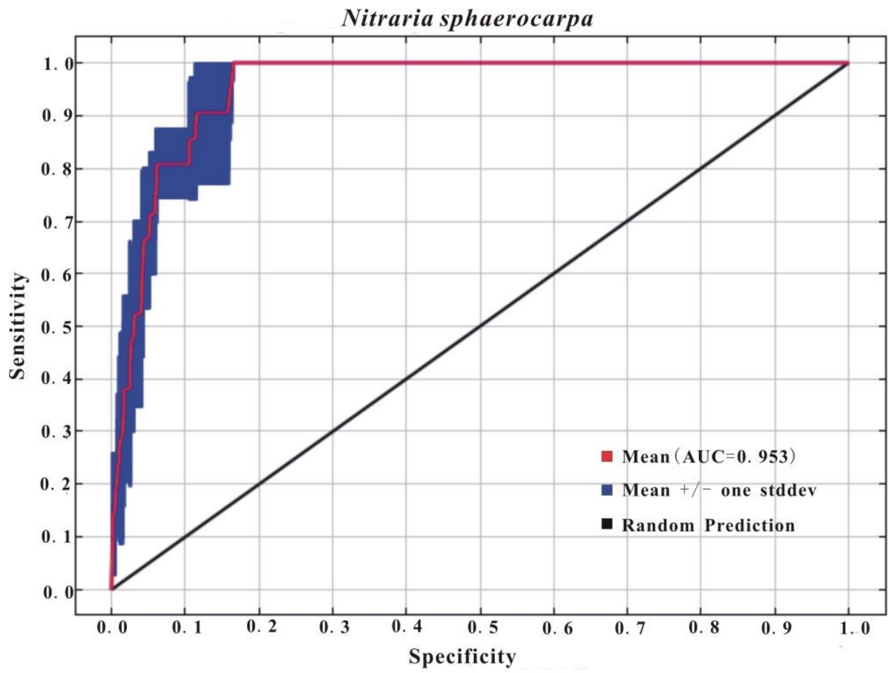

Supplementary Figure S3 ROC curve of *Nitraria tangutorum* and *N. sphaerocarpa* predicted by maximum entropy model
