# Supplementary material for: Demographic patterns of two related desert shrubs with overlapping distributions in response to past climate changes
Source: Front Plant Sci. 2024 Feb 21;15:1345624. doi: 10.3389/fpls.2024.1345624 (PMC10915042; doi:10.3389/fpls.2024.1345624)
Supplement: Supplementary file 4 [file Table_1.docx]

**Supplementary Table S1** Variable sites of the aligned sequences of two chloroplast DNA fragments (*trn*H-*psb*A and *atp*H-*atp*I) in 24 haplotypes of *Nitraria tangutorum* and *N. sphaerocarpa.* Sequences are numbered from the 5′ to the 3′ end in each region.

| **Hap** | ***trn*H-*psb*A** | | | | | | | | | | | | | | | | | | | | | | | | | | |  | ***atp*H-*atp*I** | | | | | |
| --- | --- | --- | --- | --- | --- | --- | --- | --- | --- | --- | --- | --- | --- | --- | --- | --- | --- | --- | --- | --- | --- | --- | --- | --- | --- | --- | --- | --- | --- | --- | --- | --- | --- | --- |
|  | **4**  **3** | **6**  **1** | **8**  **3** | **9**  **7** | **1**  **0**  **6** | **1**  **1**  **9** | **1**  **4**  **3** | **1**  **6**  **5** | **1**  **7**  **6** | **1**  **8**  **9** | **2**  **0**  **8** | **2**  **2**  **4** | **2**  **3**  **0** | **2**  **3**  **1** | **2**  **7**  **1** | **2**  **7**  **2** | **2**  **8**  **8** | **3**  **1**  **2** | **3**  **4**  **4** | **3**  **7**  **1** | **3**  **7**  **2** | **3**  **7**  **3** | **3**  **7**  **4** | **3**  **7**  **5** | **3**  **7**  **6** | **3**  **8**  **0** | **6**  **1**  **7** |  | **1**  **0**  **6**  **7** | **1**  **1**  **2**  **2** | **1**  **1**  **9**  **3** | **1**  **2**  **1**  **7** | **1**  **3**  **9**  **5** | **1**  **4**  **8**  **3** |
| H1 | A | G | C | T | C | A | T | A | C | C | G | G | T | A | G | C | A | G | G | - | - | - | - | - | - | A | A |  | G | C | T | G | C | A |
| H2 | A | G | C | T | C | A | T | A | A | C | G | G | T | A | G | C | A | G | G | - | - | - | - | - | - | A | A |  | G | C | T | G | C | A |
| H3 | A | G | G | G | C | A | G | A | C | C | G | G | T | A | G | C | G | T | G | - | - | - | - | - | - | A | A |  | G | C | T | G | C | C |
| H4 | A | G | G | G | C | A | T | A | C | C | G | G | T | A | G | C | G | G | G | - | - | - | - | - | - | A | A |  | G | C | T | G | C | C |
| H5 | A | A | G | G | C | A | T | A | C | C | G | G | T | A | G | C | G | G | G | - | - | - | - | - | - | A | A |  | G | C | T | G | C | C |
| H6 | A | G | G | G | C | A | T | A | C | C | G | G | T | A | G | C | G | G | A | - | - | - | - | - | - | A | A |  | G | C | T | G | C | C |
| H7 | A | G | G | G | C | A | T | A | C | C | G | A | T | A | G | C | G | G | G | - | - | - | - | - | - | A | A |  | G | C | T | G | C | C |
| H8 | A | G | G | G | C | A | T | A | C | C | G | G | T | A | G | C | G | G | A | - | - | - | - | - | - | A | G |  | G | C | T | G | C | C |
| H9 | A | G | C | G | C | A | T | A | C | C | G | G | T | A | G | C | G | G | G | - | - | - | - | - | - | A | A |  | G | C | T | G | C | C |
| H10 | A | G | G | T | C | A | T | A | C | C | G | G | T | A | G | C | G | G | G | - | - | - | - | - | - | A | A |  | G | C | T | G | C | C |
| H11 | A | G | G | T | C | A | T | A | C | C | G | G | G | A | G | C | G | G | G | - | - | - | - | - | - | A | A |  | G | C | T | G | C | C |
| H12 | A | G | G | G | C | A | T | A | C | C | T | G | T | A | G | C | G | G | G | - | - | - | - | - | - | A | A |  | G | C | T | G | C | C |
| H13 | A | G | G | G | C | A | T | A | C | A | G | G | T | A | G | C | A | G | G | - | - | - | - | - | - | A | A |  | G | C | T | G | C | C |
| H14 | A | G | G | G | C | A | T | A | C | C | G | G | T | A | G | C | G | G | G | - | - | - | - | - | - | A | A |  | G | C | T | G | A | C |
| H15 | A | G | G | G | C | A | T | A | C | C | G | G | T | A | G | A | G | G | G | - | - | - | - | - | - | A | A |  | G | C | G | G | C | C |
| H16 | A | G | G | G | T | A | T | A | C | C | G | G | T | A | G | C | G | G | G | A | A | A | A | A | A | A | A |  | G | C | T | G | A | C |
| H17 | A | G | G | G | C | A | T | A | C | C | G | G | T | A | G | A | G | G | G | - | - | - | - | - | - | A | A |  | G | A | T | A | C | C |
| H18 | A | G | G | G | C | A | T | A | C | C | G | G | T | A | G | A | G | G | G | - | - | - | - | - | - | A | A |  | G | C | T | A | C | C |
| H19 | A | G | G | G | C | A | T | A | C | C | G | G | G | A | G | C | G | G | G | - | - | - | - | - | - | A | A |  | G | C | T | G | C | C |
| H20 | A | G | G | G | C | C | T | A | C | C | G | G | T | A | G | C | G | G | G | - | - | - | - | - | - | A | A |  | G | C | T | G | C | C |
| H21 | A | G | G | G | C | C | T | A | C | C | G | G | T | C | G | C | G | G | G | - | - | - | - | - | - | A | A |  | G | C | T | G | C | C |
| H22 | A | G | G | G | C | A | T | C | C | C | G | G | T | A | G | C | G | G | G | - | - | - | - | - | - | A | A |  | G | C | T | G | C | C |
| H23 | C | G | C | G | C | A | T | A | C | C | G | G | T | A | A | A | G | G | G | A | A | A | - | - | - | C | A |  | G | C | T | G | C | C |
| H24 | A | G | G | G | C | A | T | A | C | C | G | G | T | A | G | C | G | G | G | - | - | - | - | - | - | A | A |  | A | C | T | G | A | C |
