# Supplementary material for: Demographic patterns of two related desert shrubs with overlapping distributions in response to past climate changes
Source: Front Plant Sci. 2024 Feb 21;15:1345624. doi: 10.3389/fpls.2024.1345624 (PMC10915042; doi:10.3389/fpls.2024.1345624)
Supplement: Supplementary file 5 [file Table_2.docx]

**Supplementary Table S2** Percentage of variance among populations and genetic diversity indices estimated by SAMOVA.

| Group number（K） | | Va | Vb | Vc | *F*_SC_ | *F*_ST_ | *F*_CT_ |
| --- | --- | --- | --- | --- | --- | --- | --- |
| *Nitraria tangutorum* | | | | | | | |
| 2 | 72.15 | | 14.06 | 13.78 | 0.5050 | 0.8622 | 0.7215 |
| 3 | 64.87 | | 17.33 | 17.80 | 0.4934 | 0.8220 | 0.6487 |
| 4 | 59.97 | | 10.35 | 29.69 | 0.2585 | 0.7032 | 0.5997 |
| 5 | 60.43 | | 9.28 | 30.29 | 0.2345 | 0.6971 | 0.6043 |
| 6 | 62.31 | | 4.99 | 32.70 | 0.1323 | 0.6730 | 0.6231 |
| 7 | 63.45 | | 3.41 | 33.14 | 0.0932 | 0.6686 | 0.6345 |
| 8 | 63.09 | | 3.61 | 33.29 | 0.0979 | 0.6671 | 0.6310 |
| 9 | 63.10 | | 3.00 | 33.90 | 0.0814 | 0.6610 | 0.6310 |
| 10 | 63.23 | | 2.04 | 34.73 | 0.0555 | 0.6527 | 0.6323 |
| 11 | 63.70 | | 1.12 | 35.18 | 0.0308 | 0.6482 | 0.6370 |
| 12 | 63.57 | | 0.17 | 36.27 | 0.0046 | 0.6373 | 0.6357 |
| *Nitraria sphaerocarpa* | | | | | | | |
| 2 | 81.90 | | 16.04 | 2.06 | 0.8861 | 0.9794 | 0.8190 |
| 3 | 80.08 | | 15.68 | 4.25 | 0.7868 | 0.9575 | 0.8008 |
| 4 | 87.99 | | 6.85 | 5.15 | 0.5708 | 0.9485 | 0.8799 |
| 5 | 93.75 | | 0.99 | 5.25 | 0.1593 | 0.9475 | 0.9375 |
| 6 | 94.70 | | -0.15 | 5.45 | -0.0274 | 0.9455 | 0.9470 |
| 7 | 95.18 | | -0.72 | 5.54 | -0.1501 | 0.9446 | 0.9518 |
| 8 | 95.07 | | -0.78 | 5.71 | -0.1581 | 0.9429 | 0.9507 |

Abbreviation: Va, percentage of variation among groups; Vb, percentage of variation among populations within groups; Vc, percentage of variation within populations; *F_SC_*, variance (differentiation) among populations within groups; *F_ST_*, variance (differentiation) among populations; *F_CT_*, variance among groups relative to total variance (Differentiation among groups).
