# Supplementary material for: Demographic patterns of two related desert shrubs with overlapping distributions in response to past climate changes
Source: Front Plant Sci. 2024 Feb 21;15:1345624. doi: 10.3389/fpls.2024.1345624 (PMC10915042; doi:10.3389/fpls.2024.1345624)
Supplement: Supplementary file 6 [file Table_3.docx]

**Supplementary Table S3** Group structure and diversity indices estimated by SAMOVA based on chlorotype variation.

| Group | Populations | *H* | *F*_CT_ |
| --- | --- | --- | --- |
| *Nitraria tangutorum* （K = 6） | | 0.249 | 0.6345 |
| 1 | 1 | 0.133 |  |
| 2 | 50 | 0.724 |  |
| 3 | 2,6 | 0.442 |  |
| 4 | 42,43 | 0.580 |  |
| 5 | 22,24,26,27,29,30,31 | 0.081 |  |
| 6 | 3,4,5,7,9,12,15,18,20,23,33,34,35,36,37,38,39,40,41,44,46,48,49,51,52 | 0.356 |  |
| *Nitraria sphaerocarpa* (K = 5) | | 0.099 | 0.9375 |
| 1 | 45 | 0.000 |  |
| 2 | 47 | 0.200 |  |
| 3 | 13,32 | 0.234 |  |
| 4 | 14,21,25,28 | 0.179 |  |
| 5 | 10,8,11,16,17,19, | 0.000 |  |

Abbreviation: *H*, average haplotype diversity per population in each group; *F*_CT_, differentiation among groups.
