# Supplementary material for: Demographic patterns of two related desert shrubs with overlapping distributions in response to past climate changes
Source: Front Plant Sci. 2024 Feb 21;15:1345624. doi: 10.3389/fpls.2024.1345624 (PMC10915042; doi:10.3389/fpls.2024.1345624)
Supplement: Supplementary file 7 [file Table_4.docx]

**Supplementary Table S4** Contribution rate and importance of environmental variables used for modeling

| Code | Environmental variables | *N. tangutorum* | | *N. sphaerocarpa* | |
| --- | --- | --- | --- | --- | --- |
|  |  | Contribution rate /% | Permutation importance / % | Contribution rate / % | Permutation importance  / % |
| Bio18 | Precipitation of Warmest Quarter | 49.7 | 7.5 | 51 | 0 |
| Bio4 | Temperature seasonality | 21.5 | 26 | 10.9 | 8.2 |
| Bio17 | Precipitation of driest quarter | 8 | 2.9 | 6.6 | 3.2 |
| Bio1 | Annual mean temperature | 7.8 | 14.3 | 8.9 | 6.1 |
| Bio11 | Mean temperature of coldest quarter | 7.3 | 15.4 | 4.1 | 19.3 |
| bio12 | Annual precipitation | 2.6 | 27.9 | 10.7 | 49.7 |
| bio15 | Precipitation seasonality | 0.6 | 1.3 | 5.6 | 12.8 |
