# Supplementary material for: Demographic patterns of two related desert shrubs with overlapping distributions in response to past climate changes
Source: Front Plant Sci. 2024 Feb 21;15:1345624. doi: 10.3389/fpls.2024.1345624 (PMC10915042; doi:10.3389/fpls.2024.1345624)
Supplement: Supplementary file 8 [file Table_5.docx]

**Supplementary Table S5** The potential niche areas of *Nitraria tangutorum* and *Nitraria sphaerocarpa* in different periods

**×10^4^ Km^2^**

| Species | Periods | Habitat suitability index | | | | |
| --- | --- | --- | --- | --- | --- | --- |
|  |  | 0.00-0.08 | 0.08-0.27 | 0.27-0.50 | 0.50-0.74 | 0.74-1.00 |
| *Nitraria sphaerocarpa* | LIG | 762.9720 | 66.0538 | 50.5677 | 43.9983 | 36.5156 |
|  | LGM | 798.4860 | 60.0538 | 34.6580 | 34.7170 | 30.6128 |
|  | Current | 739.3440 | 86.1692 | 60.5164 | 38.7309 | 36.1633 |
|  | Future | 802.0000 | 60.7882 | 41.2865 | 32.6632 | 24.5451 |
| *Nitraria tangutorum* | LIG | 608.9530 | 130.4390 | 78.5764 | 83.1424 | 59.9028 |
|  | LGM | 582.1810 | 157.5690 | 94.2951 | 67.5799 | 58.9965 |
|  | Current | 534.9620 | 174.9730 | 103.519 | 75.2786 | 73.3924 |
|  | Future | 547.3730 | 181.8110 | 95.7500 | 62.9566 | 72.1918 |
